# Non-occupational physical activity and risk of cardiovascular disease, cancer and mortality outcomes: a dose–response meta-analysis of large prospective studies

**DOI:** 10.1136/bjsports-2022-105669

**Published:** 2023-02-28

**Authors:** Leandro Garcia, Matthew Pearce, Ali Abbas, Alexander Mok, Tessa Strain, Sara Ali, Alessio Crippa, Paddy C Dempsey, Rajna Golubic, Paul Kelly, Yvonne Laird, Eoin McNamara, Samuel Moore, Thiago Herick de Sa, Andrea D Smith, Katrien Wijndaele, James Woodcock, Soren Brage

**Affiliations:** 1 MRC Epidemiology Unit, University of Cambridge School of Clinical Medicine, Cambridge, UK; 2 Centre for Public Health, Queen's University Belfast, Belfast, UK; 3 Singapore Institute for Clinical Sciences, Agency for Science, Technology and Research, Singapore; 4 University of Cambridge School of Clinical Medicine, Cambridge, UK; 5 Department of Medical Epidemiology and Biostatistics, Karolinska Institutet, Stockholm, Sweden; 6 Diabetes Research Centre, University of Leicester, Leicester, UK; 7 Cambridge University Hospitals NHS Foundation Trust, Cambridge, UK; 8 Physical Activity for Health Research Centre, University of Edinburgh Institute for Sport, Physical Education and Health Sciences, Edinburgh, UK; 9 Sydney School of Public Health, Prevention Research Collaboration, The University of Sydney, Sydney, New South Wales, Australia; 10 Charles Perkins Centre, The University of Sydney, Sydney, New South Wales, Australia; 11 Economic and Social Research Institute, Dublin, Ireland; 12 Center for Epidemiological Research in Nutrition and Health, University of Sao Paulo, Sao Paulo, Brazil; 13 Department of Behavioural Science and Health, University College London, London, UK

**Keywords:** epidemiology, meta-analysis, noncommunicable diseases, physical activity, health

## Abstract

**Objective:**

To estimate the dose–response associations between non-occupational physical activity and several chronic disease and mortality outcomes in the general adult population.

**Design:**

Systematic review and cohort-level dose-response meta-analysis.

**Data sources:**

PubMed, Scopus, Web of Science and reference lists of published studies.

**Eligibility criteria:**

Prospective cohort studies with (1) general population samples >10 000 adults, (2) ≥3 physical activity categories, and (3) risk measures and CIs for all-cause mortality or incident total cardiovascular disease, coronary heart disease, stroke, heart failure, total cancer and site-specific cancers (head and neck, myeloid leukaemia, myeloma, gastric cardia, lung, liver, endometrium, colon, breast, bladder, rectum, oesophagus, prostate, kidney).

**Results:**

196 articles were included, covering 94 cohorts with >30 million participants. The evidence base was largest for all-cause mortality (50 separate results; 163 415 543 person-years, 811 616 events), and incidence of cardiovascular disease (37 results; 28 884 209 person-years, 74 757 events) and cancer (31 results; 35 500 867 person-years, 185 870 events). In general, higher activity levels were associated with lower risk of all outcomes. Differences in risk were greater between 0 and 8.75 marginal metabolic equivalent of task-hours per week (mMET-hours/week) (equivalent to the recommended 150 min/week of moderate-to-vigorous aerobic physical activity), with smaller marginal differences in risk above this level to 17.5 mMET-hours/week, beyond which additional differences were small and uncertain. Associations were stronger for all-cause (relative risk (RR) at 8.75 mMET-hours/week: 0.69, 95% CI 0.65 to 0.73) and cardiovascular disease (RR at 8.75 mMET-hours/week: 0.71, 95% CI 0.66 to 0.77) mortality than for cancer mortality (RR at 8.75 mMET-hours/week: 0.85, 95% CI 0.81 to 0.89). If all insufficiently active individuals had achieved 8.75 mMET-hours/week, 15.7% (95% CI 13.1 to 18.2) of all premature deaths would have been averted.

**Conclusions:**

Inverse non-linear dose–response associations suggest substantial protection against a range of chronic disease outcomes from small increases in non-occupational physical activity in inactive adults.

**PROSPERO registration number** CRD42018095481.

## Introduction

Cardiovascular disease (CVD) is the leading cause of death globally, responsible for 17.9 million annual deaths in 2019,^1^ whereas cancers were responsible for 9.6 million deaths in 2017.^1^ Both conditions top the global disease burden with respect to disability-adjusted life-years.^2^ The relative contribution of various risk and protective factors to the incidence of, and mortality from, these conditions is an ongoing debate.

Higher levels of physical activity (PA) are associated with lower risk of all-cause mortality, CVD and some site-specific cancers.^2–8^ The highest quality evidence for these associations and the population impact of low PA levels comes from disease-specific meta-analyses. However, differing methods used in previous meta-analyses limit comparability of relative risks across different outcomes. The only initiative to consistently consider the population impact across diseases (five in total) is the Global Burden of Disease (GBD) study, which uses estimates of total PA, including occupational activity.^9^ This is problematic as occupational activity is generally poorly measured compared with non-occupational activity and estimates of activity at work often dwarf the non-occupational component. One reason for this is the use of gross metabolic equivalent of task (MET)-hour/week to quantify PA volume. Sedentary office work may be considered to cost approximately 1.25 METs, of which 1 MET represents resting metabolic rate. Non-occupational activity is typically more intense (eg, walking at 4.0 km/hour is approximately 3 METs), meaning the relative contribution of the resting metabolic rate is smaller. Including low-intensity high-duration activities such as occupational activity without marginalising the resting component can therefore distort the PA exposure dramatically. Furthermore, even when occupational PA is well measured, it remains unclear whether it has similar health benefits to non-occupational activity.^10^ These factors add statistical noise, decreasing the accuracy of the estimated associations, and may underestimate the contribution of non-occupational PA.

Accurate estimation of the dose–response association between PA and disease outcomes, combined with prevalence estimates, is a prerequisite to assessing the population disease burden of insufficient PA and the potential impact of changes to population levels of PA. Modelling studies that evaluate public health strategies aimed at increasing population activity levels have found that the shape of the dose–response association makes a significant difference when quantifying population health impacts.^6^

Our objective was to quantify dose–response associations between non-occupational PA and several CVD, cancer and mortality outcomes using a novel harmonisation framework to overcome the challenges posed by different PA measurement methods. This framework allowed us to compare studies measuring and reporting PA in a wide variety of ways on the same activity exposure scale of marginal MET-hours per week (mMET-hours/week). This meant we could be more inclusive of the available evidence than previous meta-analyses and we could explore the dose–response relationships between non-occupational PA and nine site-specific cancers for the first time. To contextualise our results for public health promotion, we generated potential population impact fractions (PIFs) to estimate the proportion of preventable deaths and disease outcomes at different non-occupational PA levels.

## Methods

The study protocol is available at PROSPERO (registration number CRD42018095481).

### Eligibility criteria

Online supplemental eMethods 1 shows the study inclusion and exclusion criteria. We included prospective cohort studies that followed adults (≥18 years) without pre-existing conditions, reported PA at baseline in at least three ordinal exposure levels and reported risk estimates for the examined outcomes. We excluded studies with <10 000 participants to limit potential bias from small study sizes with positive results; this is the required cohort sample size for outcomes with up to 70% of subjects in the unexposed group (ie, no or less physically active), expected incidence rate in the unexposed group around 15% or more, and assumed relative risk of 0.85, when type I and type II errors are 0.05 and 0.20, respectively. These are reasonable parameters for the investigated populations, exposure and endpoints. We also excluded studies where the follow-up period was <3 years to minimise reverse causality bias, which is in accordance with the findings of Lee *et al*.^11^

10.1136/bjsports-2022-105669.supp1 Supplementary data

Only articles that examined leisure-time PA, alone or in combination with other domains or specific types of activity, were included. We excluded articles whose measures of PA contained occupational activity that could not be factored out and that investigated individual domains of PA that did not include leisure-time activity (eg, transport-related PA only).

The outcomes of interest were the following:

- All-cause, total CVD and total cancer mortality.
- Total CVD, coronary heart disease, stroke and heart failure incidence (fatal and non-fatal events combined).
- Total and site-specific (head and neck, myeloid leukaemia, myeloma, gastric cardia, lung, liver, endometrial, colon, breast, bladder, rectum, oesophageal, prostate, and kidney) cancer incidence (fatal and non-fatal events combined). These site-specific cancers were selected based on previously reported associations with PA.^8^

### Search and selection process

We searched PubMed, Scopus, Web of Science and the reference lists of reviews retrieved from our systematic review, known to the authors or cited in the 2018 US Physical Activity Guidelines Advisory Committee Scientific Report.^12^ Online supplemental eMethods 2 details the systematic search strategy. We considered peer-reviewed articles in any language published in academic journals until February 2019.

Titles and abstracts, and subsequently full texts, were screened independently twice for eligibility (online supplemental eFigure 1). Disagreements were resolved by discussion.

If multiple articles reported results on the same cohort and outcome, we followed specific criteria (online supplemental eMethods 3) to select one.

### Data extraction

Data from each paper were extracted by one reviewer and double-checked independently by a second reviewer. Disagreements were checked against the full text and resolved by discussion.

We extracted data on publication (first author, year of publication), study characteristics (country, cohort name, sample size, age and sex of participants, and duration of follow-up), PA exposure assessment (instrument, domains of activity and exposure categories) and outcome assessment (defined using the International Classification of Disease 10th revision (ICD-10) codes as reported in the PROSPERO registration and online supplemental eMethods 1). We extracted whether fatal, non-fatal or the combination of fatal and non-fatal events was tallied. We also identified how original study analyses had considered baseline morbidity (exclusion at baseline or statistical adjustment in multivariable regression models) and early incident cases during follow-up.

For each exposure category, we extracted information to quantify PA volume, number of cases, number of participants and/or person-years of follow-up, and risk estimates with 95% CIs. Risk estimates from the most adjusted model were used. When available, risk estimates of the most adjusted model without adiposity-related covariates (eg, body mass index (BMI), waist circumference) were retrieved and used in sensitivity analyses. Exposure data were extracted to the level of precision reported in the published study before being harmonised to PA volume in mMET-hours/week. Risk estimates were extracted exactly as reported. Results reported separately by sex or other attributes (eg, age, ethnicity) or for multiple cohorts within an article were treated as separate associations. If the original publication did not fully report information required for the meta-analysis, data were obtained from other publications using the same cohort, imputation procedures (online supplemental eMethods 4) or by contacting the authors. We had no control over the methods used to handle confounders and missing data in the original analyses.

### PA exposure harmonisation

#### Overview of harmonisation

A comprehensive data harmonisation was conducted to combine PA data from different sources. We harmonised reported PA exposure levels from all included studies into a common metric of non-occupational PA volume in mMET-hours/week, reflecting the rate of energy expenditure outside of work, above the resting metabolic rate (1 MET). This allows correct equating of PA volume of time spent at different PA intensity levels.^13^ The principles of each aspect of our harmonisation procedure are described in the following sections and presented in a flow chart available in our OSF repository. The last columns of the table on ‘Details of included articles and original and harmonized exposures’ in the same OSF repository contain the details of how the original PA exposure categories of each study were harmonised to mMET-hours/week values.

#### Frequency, duration and intensity assumptions

PA exposures described in studies as frequency and duration measures were converted to weekly duration. If session duration was not provided, we assumed a duration of 0.75 hours/session (0.5 hours/session in sensitivity analyses). Categorical frequency data (eg, never, sometimes and often) were converted to weekly duration using assumptions for both frequency (eg, 0, 2 and 5 sessions per week) and duration of sessions. When PA intensity was not explicitly described, we considered the reported activities as light, moderate or vigorous based on the description provided and using the Compendium of Physical Activities.^14^ We assigned mMET values of 1.5 for light, 3.5 for moderate and moderate-to-vigorous, and 7.0 for vigorous PA (1 mMET less in sensitivity analyses).

#### Converting absolute energy expenditure to MET values

For studies reporting energy expenditure without adjustment for body weight (eg, kJ/day, kcal/week), energy expenditure was divided by reported weights to derive MET equivalents (1 MET=1 kcal/kg/hour). If unavailable, body weight was calculated from reported BMI and height. If BMI was reported without height, a mean value from national survey data was used for height.

#### Subtracting resting energy expenditure from estimates of PA

To marginalise studies reporting volume of PA in gross units, 1 MET-hour was subtracted for each hour of activity. If the mean duration was not available, we used a conversion equation derived from all remaining studies where both volume and duration were available (online supplemental eMethods 5).

#### Isolating the non-occupational component of aggregate PA estimates

Some articles provided aggregated exposures of non-occupational and occupational PA alongside other behaviours, such as sleep or sedentary time. When quantified information about these behaviours was available, this was subtracted from the point estimate of each exposure category. If no quantifiable data were available, we assumed occupational activity to be 40 hours/week at 1.25 MET (or 0.25 mMET) or the value of the lowest exposure category and subtracted this from all exposure categories.

### Meta-analytical methods

We conducted a meta-analysis for any outcome with results from at least four cohorts. Where necessary, risk estimates were recalculated to set the least active category as the referent.^15^ For studies reporting only stratified results (eg, sex or ethnicity), we combined stratum-specific risks into overall population estimates using fixed-effect meta-analysis.

We performed a two-stage random-effects meta-analysis. In the first stage, we estimated study-specific dose–response coefficients using generalised least squares to incorporate the correlation within each set of log-relative risks.^16 17^ In the second stage, we estimated the pooled coefficients by combining study-specific dose–response coefficients using restricted maximum likelihood,^18 19^ with cohorts weighted by the inverse of their variance. We assumed non-linearity of dose–response associations,^2–4 7 9 13^ and thus modelled them by fitting restricted cubic splines. Given that the volumes of PA reported in most studies were at the lower end of the exposure range and that there is greater uncertainty about the reliability of very high levels of self-reported PA, we set three knots at the 0th, 37.5th and 75th percentiles of person-years rather than persons (0th, 42.5th and 85th percentiles in sensitivity analyses). The slope was fixed at the last knot. If the statistical model was unable to converge, we progressively increased the percentile for the upper knot by 1% until model convergence. Dose–response curves were drawn from 1000 points evenly distributed between 0 mMET-hour/week and the largest PA dose observed in the cohorts included for the outcome of interest.

To investigate the potential effect of study-level confounders on the pooled results across outcomes, we conducted subgroup analyses with the 11 studies that reported results for all all-cause, CVD and cancer mortality outcomes and contrasted them with results from the corresponding main analysis. We also conducted subgroup analysis by sex using studies that reported separate results for men and women.

### Estimation of population health impact

PIFs were calculated for all outcomes based on PA exposure levels in the population of all included cohorts for a given outcome (see formula in online supplemental eMethods 6).^20^ PIFs were calculated for three exposure levels based on the equivalents of the WHO moderate-to-vigorous aerobic PA recommendations for adults^21^: 8.75 mMET-hours/week (the minimum recommended level, equivalent to 2.5 hours/week of PA at an intensity of 3.5 mMET, such as brisk walking), 17.5 mMET-hours/week (upper bound of recommended levels for health benefits) and 4.375 mMET-hours/week (half the minimum recommended level). The risk estimates used to calculate PIFs were taken from the dose–response curves, which pooled the most adjusted associations provided by the individual studies.

### Risk of bias assessment

We explored the impact of six potential sources of bias in individual articles and our meta-analytical procedures: how studies had analysed participants with other morbidities, whether they excluded early incident cases during follow-up, duration of follow-up, imputation procedures for missing data, aspects of our approach to exposure harmonisation and separation of occupational PA. For each of these, we contrasted the overall risk estimates between groups of studies with different characteristics. This was done for the five outcomes with the largest number of separate results (all-cause mortality, and total CVD and cancer mortality and incidence), using a fixed PA level of 8.75 mMET-hours/week, relevant to the six sources of bias.

### Software, data and code availability

Analyses were performed using R V.4.0.5 and the *dosresmeta* package (V.2.0.1).^19^ An interactive interface to visualise the dose–response associations was developed using the Shiny package (V.1.0.5). Syntax for all analyses and the interactive interface are available at https://github.com/meta-analyses/.

### Patient and public involvement

Patients and the public were not involved in the development of this work.

## Results

### Identified literature

We screened titles and abstracts of 48 525 articles, of which 1280 were selected for full-text screening. A total of 196 articles^2 22–216^ were included in the meta-analysis (online supplemental eFigure 1), covering 94 cohorts and reporting 330 separate results. The evidence base was largest for all-cause mortality (50 results; 163 415 543 person-years, 811 616 events), total CVD incidence (37 results; 28 884 209 person-years, 74 757 events) and total cancer incidence (31 results; 35 500 867 person-years, 185 870 events). Details of all selected and excluded articles can be found in our OSF repository, and data underlying each of the dose–response estimations can be downloaded at https://shiny.mrc-epid.cam.ac.uk/meta-analyses-physical-activity /.

### Primary dose–response analyses

**Figure 1:**
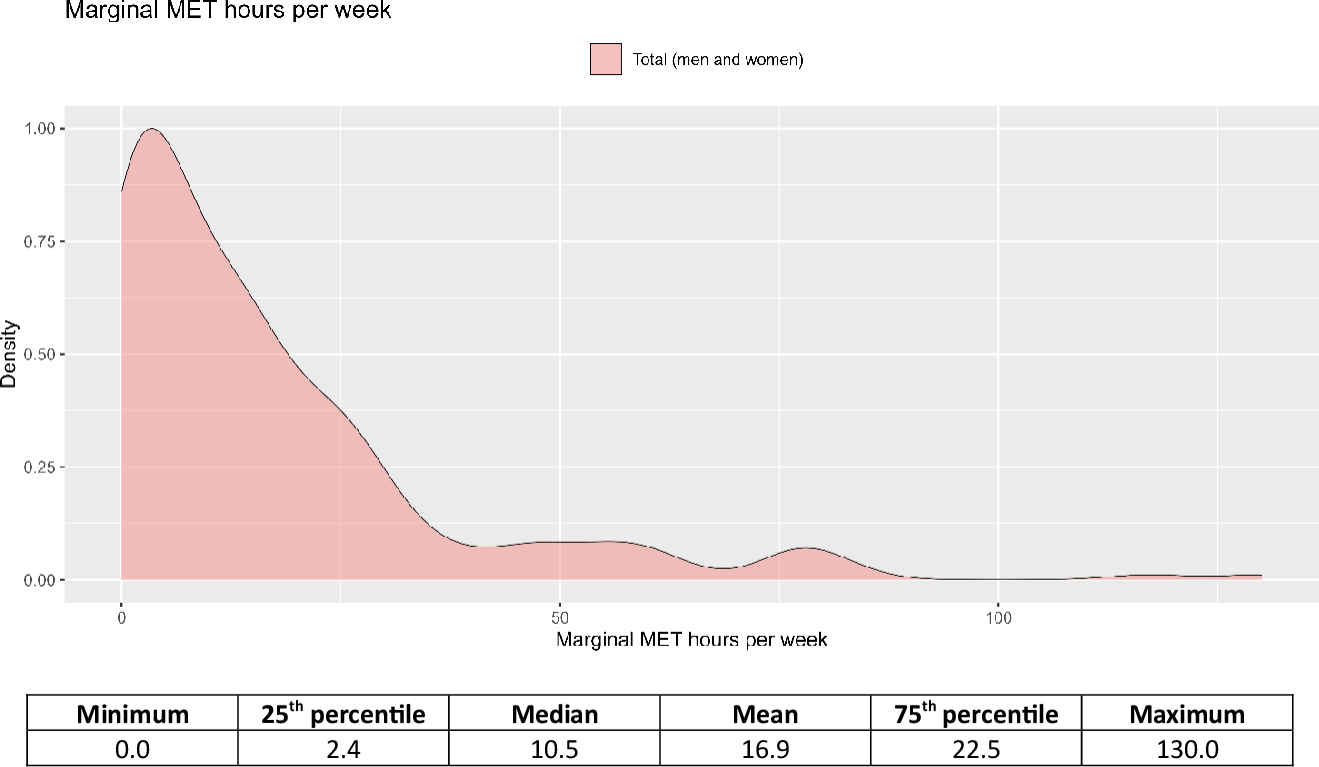
Distribution of marginal MET-hours/week for cohorts included in the all-cause mortality analysis. The exposure distribution for cohorts included in the analysis of other outcomes is available at https://shiny.mrc-epid.cam.ac.uk/meta-analyses-physical-activity /. MET, metabolic equivalent of task.

Most study participants reported non-occupational PA levels below 17.5 mMET-hours/week (76% of person-years), with almost all data below 35 mMET-hours/week (94% of person-years). Figure 1 shows the exposure distribution for the cohorts included in the all-cause mortality analysis.

Inverse, curvilinear dose–response associations between PA and most outcomes were observed, with stronger associations at lower volumes of PA. In most cases, diminishing marginal differences in risk and increasing uncertainty were observed at high PA volumes, particularly beyond 17.5 mMET-hours/week. Interactive dose–response curves and the exposure distributions are available at https://shiny.mrc-epid.cam.ac.uk/meta-analyses-physical-activity /.

**Figure 2:**
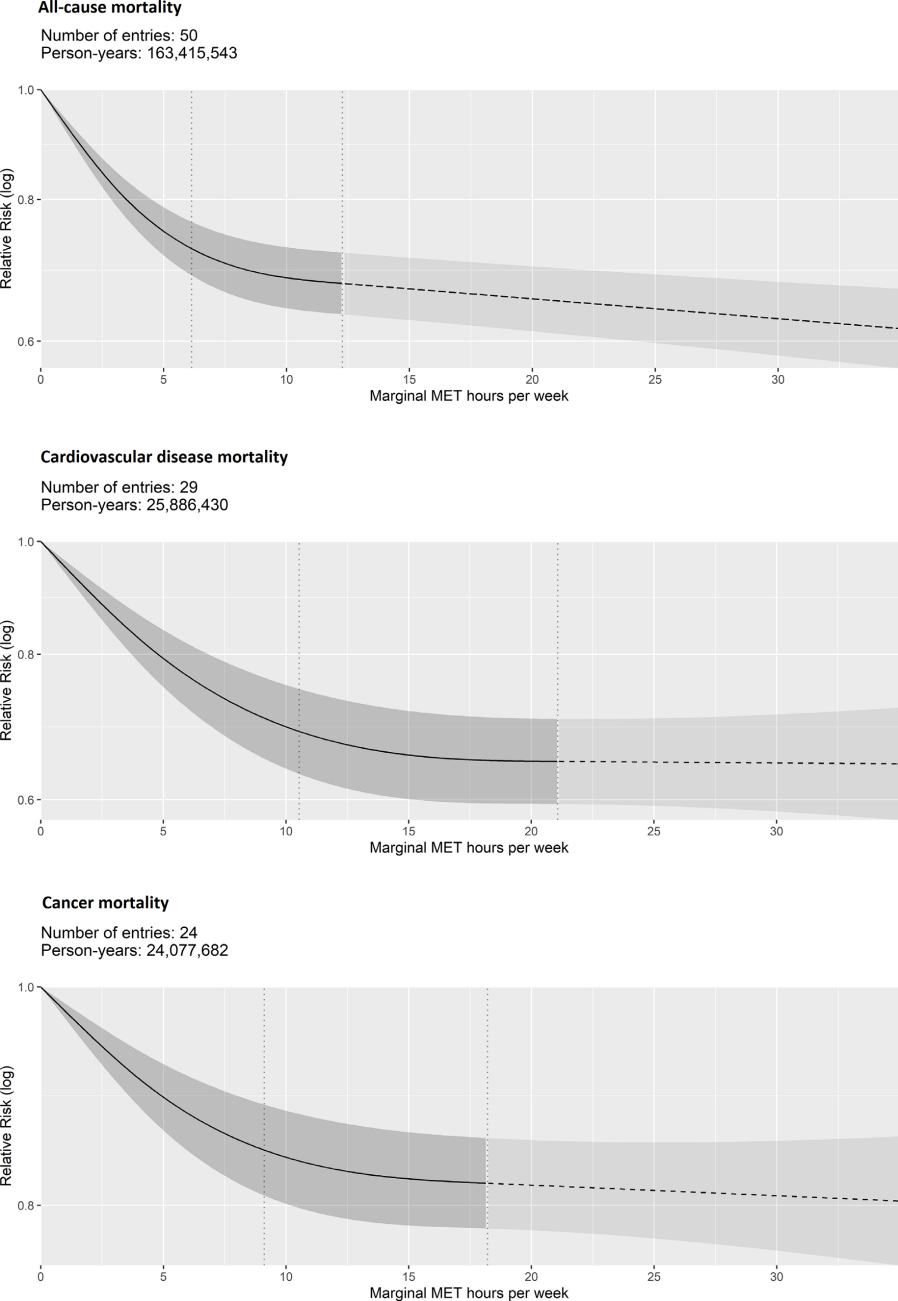
Dose–response association between non-occupational physical activity and mortality outcomes. Vertical dashed lines: cubic spline knots (0th, 37.5th and 75th percentiles of person-years); dashed line beyond the upper knot: constrained to be linear. MET, metabolic equivalent of task.

The association was stronger and more curvilinear for all-cause and CVD mortality than for cancer mortality (figure 2, table 1). Compared with inactive individuals, adults accumulating 8.75 mMET-hours/week had 31% (95% CI 27 to 35) and 29% (95% CI 23 to 34) lower risk of all-cause and CVD mortality, respectively, whereas the risk difference for total cancer mortality was 15% (95% CI 11 to 19).

**Table 1:** Relative risk of mortality and incidence of cardiovascular diseases and cancers at three physical activity levels in relation to 0 mMET-hours/week and respective potential impact fractions

Outcomes	4.375 mMET-hours/week		8.75 mMET-hours/week		17.5 mMET-hours/week	
	RR (95% CI)	PIF (95% CI)	RR (95% CI)	PIF (95% CI)	RR (95% CI)	PIF (95% CI)
Mortality						
All-cause mortality	0.77 (0.73 to 0.80)	10.11 (8.32 to 11.91)	0.69 (0.65 to 0.73)	15.66 (13.16 to 18.15)	0.66 (0.62 to 0.70)	18.75 (16.09 to 21.39)
Total CVD	0.81 (0.77 to 0.85)	6.02 (4.42 to 7.69)	0.71 (0.66 to 0.77)	12.25 (9.37 to 15.17)	0.65 (0.60 to 0.71)	17.05 (13.92 to 20.19)
Total cancer	0.90 (0.88 to 0.93)	3.68 (2.54 to 4.86)	0.85 (0.81 to 0.89)	7.05 (5.1 to 9.00)	0.82 (0.78 to 0.86)	9.29 (7.36 to 11.22)
CVD incidence (fatal and non-fatal events combined)						
Total CVD	0.83 (0.79 to 0.87)	5.29 (3.93 to 6.70)	0.73 (0.69 to 0.79)	10.86 (8.43 to 13.31)	0.67 (0.63 to 0.72)	15.42 (13.01 to 17.85)
Coronary heart disease	0.86 (0.83 to 0.90)	5.51 (3.95 to 7.11)	0.79 (0.74 to 0.84)	10.28 (7.61 to 12.96)	0.74 (0.69 to 0.80)	13.58 (10.51 to 16.64)
Stroke	0.86 (0.83 to 0.90)	5.53 (3.96 to 7.15)	0.80 (0.75 to 0.85)	9.77 (7.31 to 12.24)	0.77 (0.72 to 0.82)	12.37 (9.63 to 15.10)
Heart failure	0.90 (0.85 to 0.96)	2.23 (0.80 to 3.89)	0.84 (0.75 to 0.93)	4.63 (1.90 to 7.66)	0.77 (0.68 to 0.87)	7.86 (4.31 to 11.68)
Cancer incidence (fatal and non-fatal events combined)						
Total cancer	0.93 (0.91 to 0.95)	2.53 (1.66 to 3.42)	0.88 (0.85 to 0.92)	5.22 (3.55 to 6.90)	0.85 (0.81 to 0.89)	7.80 (5.65 to 9.93)
Head and neck	0.74 (0.59 to 0.94)	10.59 (2.10 to 19.96)	0.65 (0.46 to 0.91)	17.46 (3.68 to 30.98)	0.63 (0.42 to 0.94)	19.83 (1.23 to 36.55)
Myeloid leukaemia	0.80 (0.66 to 0.97)	4.93 (0.68 to 9.70)	0.75 (0.60 to 0.95)	7.08 (1.65 to 12.94)	0.75 (0.60 to 0.94)	7.57 (2.23 to 13.33)
Myeloma	0.84 (0.74 to 0.95)	3.34 (0.90 to 6.26)	0.75 (0.61 to 0.92)	6.76 (1.90 to 12.09)	0.72 (0.57 to 0.92)	8.30 (1.91 to 14.99)
Gastric cardia	0.86 (0.78 to 0.95)	1.95 (0.58 to 3.68)	0.78 (0.66 to 0.91)	4.77 (1.58 to 8.57)	0.73 (0.60 to 0.88)	6.77 (2.55 to 11.63)
Lung	0.89 (0.87 to 0.92)	4.39 (3.16 to 5.63)	0.84 (0.81 to 0.88)	7.26 (5.10 to 9.40)	0.83 (0.76 to 0.91)	8.54 (3.04 to 13.98)
Liver	0.90 (0.83 to 0.98)	3.49 (0.58 to 6.71)	0.84 (0.73 to 0.96)	6.75 (1.44 to 12.31)	0.78 (0.66 to 0.93)	10.27 (3.26 to 17.31)
Endometrial	0.94 (0.90 to 0.99)	1.10 (0.26 to 2.02)	0.90 (0.84 to 0.97)	2.62 (0.76 to 4.60)	0.87 (0.80 to 0.95)	4.54 (1.88 to 7.28)
Colon	0.96 (0.93 to 0.99)	0.70 (0.11 to 1.33)	0.93 (0.87 to 0.99)	1.69 (0.33 to 3.12)	0.90 (0.84 to 0.97)	2.96 (0.92 to 5.05)
Breast	0.97 (0.96 to 0.99)	0.69 (0.32 to 1.07)	0.95 (0.92 to 0.97)	1.62 (0.79 to 2.46)	0.92 (0.88 to 0.96)	3.23 (1.85 to 4.63)
Bladder	0.93 (0.84 to 1.02)	1.91 (−0.54 to 4.52)	0.90 (0.80 to 1.02)	2.98 (−0.24 to 6.33)	0.90 (0.81 to 1.01)	3.11 (0.02 to 6.35)
Rectum	0.96 (0.92 to 1.01)	0.77 (−0.15 to 1.75)	0.95 (0.88 to 1.02)	1.43 (−0.45 to 3.36)	0.96 (0.88 to 1.04)	0.52 (−1.74 to 2.82)
Oesophageal	0.97 (0.89 to 1.05)	0.68 (−0.95 to 2.68)	0.95 (0.82 to 1.09)	1.38 (−2.06 to 5.32)	0.94 (0.79 to 1.12)	1.57 (−3.05 to 6.67)
Prostate	1.00 (0.99 to 1.02)	−0.10 (−0.48 to 0.29)	1.01 (0.98 to 1.04)	−0.21 (−1.10 to 0.71)	1.01 (0.96 to 1.05)	−0.10 (−1.61 to 1.42)
Kidney	1.02 (0.92 to 1.13)	−0.65 (−3.69 to 2.81)	1.03 (0.89 to 1.19)	−1.23 (−6.39 to 4.34)	1.04 (0.88 to 1.24)	−1.91 (−9.20 to 5.63)

CVD, cardiovascular disease; mMET, marginal metabolic equivalent of task; PIF, potential impact fraction; RR, relative risk.

**Figure 3:**
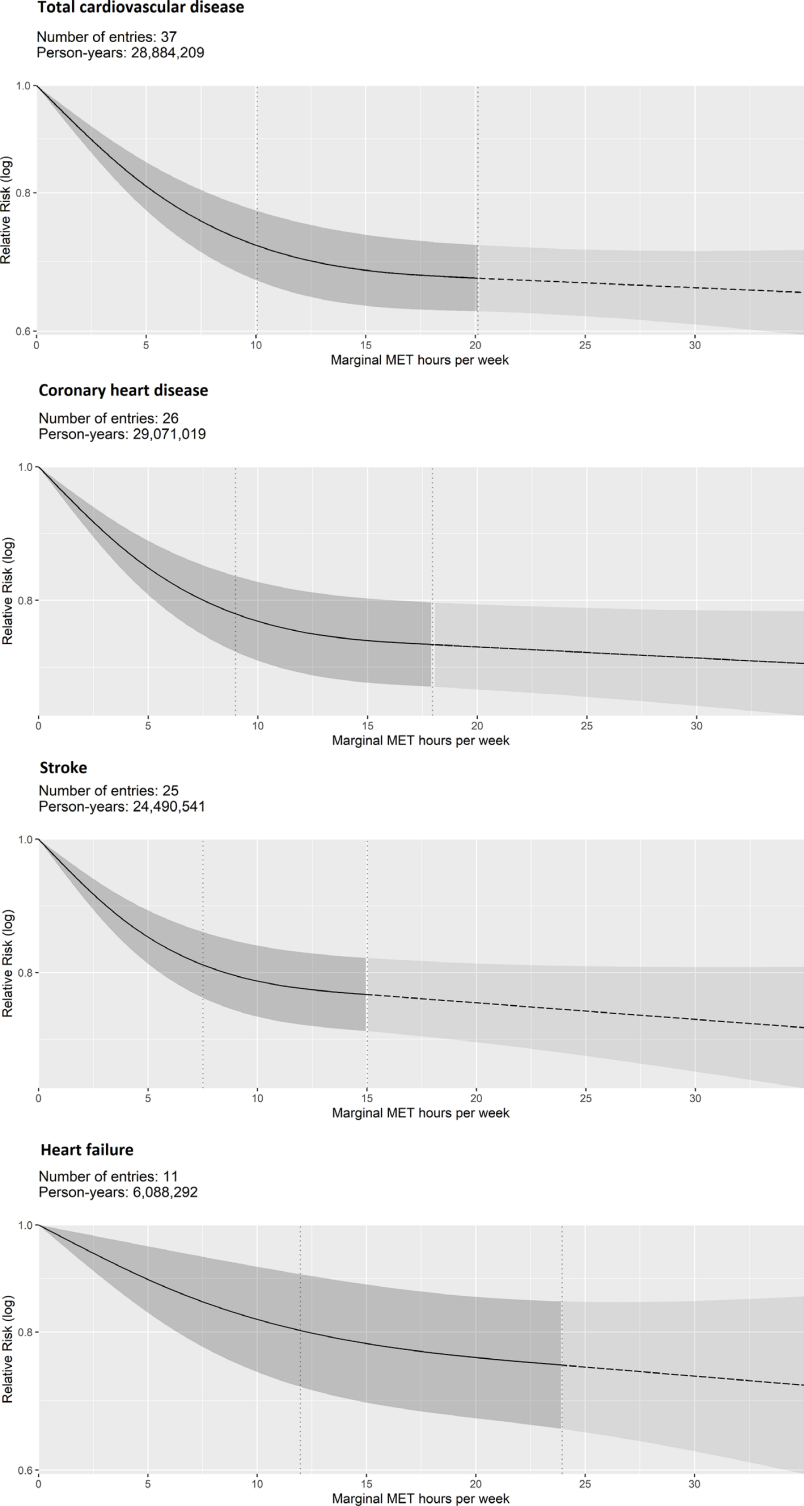
Dose–response association between non-occupational physical activity and incidence (fatal and non-fatal events combined) of cardiovascular diseases. Vertical dashed lines: cubic spline knots (0th, 37.5th and 75th percentiles of person-years); dashed line beyond the upper knot: constrained to be linear. MET, metabolic equivalent of task.

A strong curvilinear association was observed for total CVD incidence (27% lower risk (95% CI 21 to 31) at 8.75 mMET-hours/week). However, the associations were weaker and more linear for the incidence of specific CVD outcomes (coronary heart disease, heart failure and stroke), with the strongest association observed for coronary heart disease (21% lower risk (95% CI 16 to 26) at 8.75 mMET-hours/week) (figure 3, table 1).

**Figure 4:**
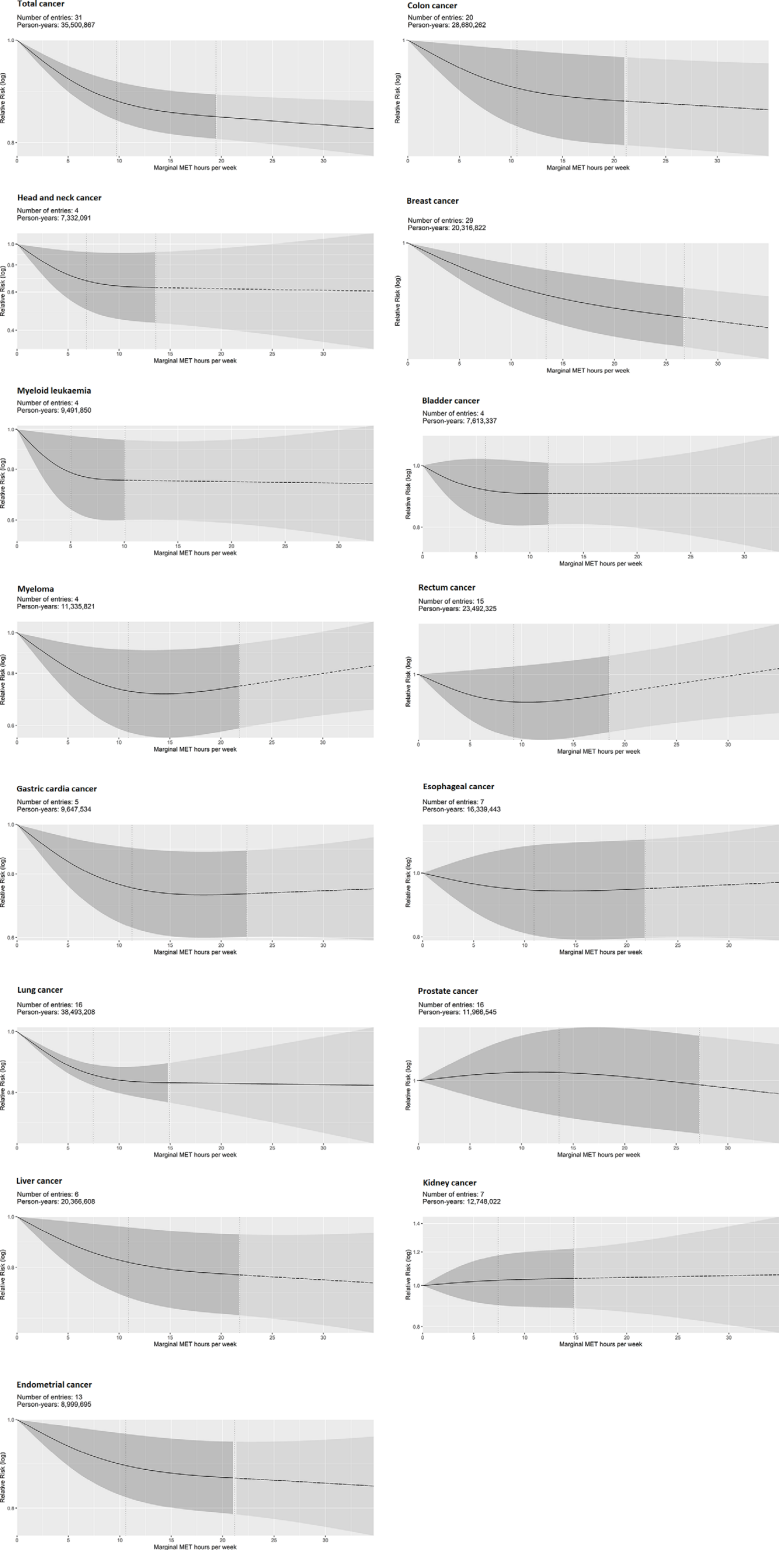
Dose–response association between non-occupational physical activity and incidence (fatal and non-fatal events combined) of cancers. Vertical dashed lines: cubic spline knots (0th, 37.5th and 75th percentiles of person-years); dashed line beyond the upper knot: constrained to be linear. MET, metabolic equivalent of task.

The association was weaker and more linear for total cancer incidence (12% lower risk (95% CI 8 to 15) at 8.75 mMET-hours/week). For the incidence of site-specific cancers, curvilinear and stronger associations were observed for head and neck, myeloid leukaemia, myeloma, and gastric cardia (from 35% to 22% lower risk at 8.75 mMET-hours/week). Weaker and more linear associations were observed for lung, liver, endometrial, colon and breast (from 16% to 5% lower risk at 8.75 mMET-hours/week). Non-significant associations were observed for bladder, oesophageal, prostate and rectal cancer (figure 4, table 1). No eligible studies were available for malignant melanoma.

### Potential population impact

Assuming a causal relationship between non-occupational PA and the examined health outcomes, shifting the distribution of non-occupational PA in the cohorts towards the equivalent recommended level of moderate-to-vigorous aerobic activity (8.75 mMET-hours/week) would have averted a substantial fraction of the population disease burden. For example, if all individuals accumulated at least 8.75 mMET-hours/week, then 15.7% (95% CI 13.2 to 18.2), 12.3% (95% CI 9.4 to 15.2) and 7.1% (95% CI 5.1 to 9.0) of all-cause, CVD-related and cancer-related deaths, respectively, potentially would have been averted. Additionally, 10.9% (95% CI 8.4 to 13.3) and 5.2% (95% CI 3.6 to 6.9) of all incident cases of CVD and cancer would have been prevented. Notably, 10.1% (95% CI 8.3 to 11.9) of all deaths would have been prevented if all adults achieved at least half this PA level (table 1).

### Subgroup analysis

#### Pooled estimates from studies that reported results for all-cause, CVD and cancer mortality

The pooled analysis from the 11 studies that reported results for all-cause, CVD and cancer mortality showed weaker associations for all-cause mortality and stronger associations for CVD and cancer mortality (online supplemental eResults 1).

#### Sex-stratified results

Sex-stratified results were available for all-cause, CVD and cancer mortality and incidence of total CVD, coronary heart disease, stroke, total cancer, and cancers of the colon and rectum. There was some evidence of stronger associations in women than in men for all-cause and CVD mortality and CVD incidence, but the reverse for cancer mortality and incidence (online supplemental eResults 2).

### Risk of bias

Across the five outcomes with the largest number of separate results (all-cause mortality, and total CVD and cancer mortality and incidence), 83%–93% of the studies used statistical adjustments to control for other morbidities at baseline, often combined with exclusion of adults with more serious pre-existing conditions. Studies that predominantly used statistical adjustments tended to report stronger associations for CVD mortality compared with studies that excluded adults with pre-existing conditions at baseline. Between 54% and 59% of the studies did not exclude mortality and disease events occurring within the first few years of follow-up, and those that did tended to report weaker associations for all-cause mortality and CVD mortality and incidence. The median follow-up time was 10 years for each outcome, with no evidence of bias between studies with longer and shorter follow-up time. Data imputation was required for 58%–64% of the studies, but no evidence of bias was found. The most frequently used procedures for exposure harmonisation were measurement unit conversions (42%–49% of studies) and the assumption of standard values for the intensity and duration of PA reported (39%–50%), but no evidence of bias was found. In 75% of the studies, the original exposure assessment did not include occupational PA and no evidence of bias was observed for studies requiring domain separation (online supplemental eResults 3).

### Sensitivity analyses

In studies that did not report the duration or intensity of PA, assuming (where appropriate) shorter session duration (0.5 hours instead of 0.75 hours) and lower intensity (1 mMET lower for moderate and vigorous PA) did not materially alter the dose–response associations for most outcomes, especially analyses which included the largest number of separate results (online supplemental eResults 4). When shorter duration and lower intensity were used, associations were stronger for CVD outcomes, particularly at lower PA volumes. Placing the restricted cubic spline knots at the 42.5th and 85th percentiles resulted in less stable estimations at higher PA volumes, with those specific segments of the dose–response curves being based on less data and with a higher risk of exposure misclassification.

Sensitivity analyses using analytical models that did not include adjustment for adiposity showed stronger associations for all-cause and CVD mortality, and the incidence of total CVD, heart failure, and site-specific cancers of the endometrium, kidney, lung and rectum (online supplemental eResults 4). Notably, the association was significant for rectal cancer.

## Discussion

We present comprehensive dose–response meta-analyses of the associations between non-occupational PA and a wide range of mortality and disease outcomes. Our extensive exposure data harmonisation allowed inclusion of a larger evidence base than previous meta-analyses for 17 of 22 health outcomes investigated. For instance, our meta-analysis for all-cause mortality includes over 160 million person-years and 810 000 deaths. These are 17 and 7 times larger, respectively, than what were included in the previously largest dose–response analysis to date (9.39 million person-years and 117 000 deaths).^2^ Our inclusive approach has a number of advantages. First, we were able to estimate dose–response associations for nine site-specific cancers for the first time. Second, inclusion of more data increases confidence in the results beyond previous estimates. Third, it allowed us to include data from a larger number of countries. Fourth, having used consistent methods for several health outcomes allows the best possible comparison of the shape of the dose–response functions across these outcomes. For example, having one set of dose–response results consistently estimated across several outcomes is particularly useful for health impact modelling, for example, to quantify the probable impacts of changes to population-level PA.

We showed inverse non-linear dose–response associations for all-cause mortality and a range of CVD and cancer outcomes. This suggests that the greatest potential benefits can be achieved through small increases in non-occupational PA for those with an inactive lifestyle, with incrementally smaller additional benefits up to approximately 17.5 mMET-hours/week. Above this level, the evidence base was weaker. Shifting population levels of activity towards achieving the equivalent to 150 min/week of moderate-to-vigorous aerobic PA (8.75 mMET-hours/week) potentially would have prevented 16% of all premature deaths recorded.

Compared with previous studies, we found similar associations for all-cause^2 3^ and cancer mortality,^4^ and stronger associations and narrower CIs for CVD mortality,^5 6^ at the equivalent recommended levels of moderate-to-vigorous aerobic PA. For cancers of the bladder, breast, colon, endometrium, oesophagus, liver and rectum, our results were largely similar to that of a recent pooled analysis.^7^ However, we found stronger associations for cancers of the gastric cardia, head and neck, myeloid leukaemia, and myeloma, whereas the association for kidney cancer was non-significant. Contrasting with Moore *et al*’s^8^ finding of harmful effects of PA on prostate cancer, our results did not show an association. Comparisons should be interpreted with caution as most previous studies have focused solely on leisure-time PA^2 3 6–8^ and meta-analyses by Li *et al* 4 and Wahid *et al* 5 included studies that assessed PA in any domain.

Compared with the results from the GBD,^9^ which has assessed total PA rather than non-occupational PA, we found stronger associations for heart disease and stroke at the equivalent minimum recommended moderate-to-vigorous aerobic PA level. Associations reported by the GBD at approximately 130 MET-hours/week for total PA were observed in our study at 17.5 mMET-hours/week for non-occupational PA, a much lower volume even considering that we excluded energy expenditure from the resting metabolic rate and occupational activity. Given the challenges of assessing occupational activity, estimates of total PA from self-report are often implausibly high.^217^ Hence, the importance of PA may have been underestimated by the GBD. This, combined with uncertainties around the health benefits of occupational PA,^10 218^ means our results may be more relevant from a public health perspective.

It is surprising that the risk difference for all-cause mortality (31% at the equivalent minimum recommended moderate-to-vigorous aerobic PA level) is similar to that for total CVD (29%) and much larger than for total cancer mortality (15%). Equally, it is surprising that the association for total CVD incidence is stronger than that for coronary heart disease or stroke. Weaker associations would be expected for more composite outcomes (eg, all-cause mortality and total CVD incidence) than for the outcomes within them for which a significant association has been established. Potential explanations include detection bias for some disease events, misclassification of causes of death (especially for more specific disease outcomes), differential residual confounding, reverse causality by outcome, and different inclusion criteria by disease outcome, such as the inclusion of different disease groups which may have stronger associations with PA.^219^ There may also be study-level confounders, and this is supported by our post-hoc subgroup analysis, which showed that studies reporting all three mortality outcomes found stronger associations for CVD than for all-cause mortality. Taken together, it is probable that the risk estimates for all-cause mortality are more accurate than for specific disease outcomes.

Our risk of bias and sensitivity analyses showed that the methodological assumptions and decisions made for the pooled analyses did not significantly impact the dose–response associations observed. However, some methodological differences between the original studies seem to alter the magnitude of the pooled associations. Studies that did not exclude people with pre-existing conditions at baseline or mortality or disease events occurring within the first years of follow-up tended to observe stronger associations, as did studies with shorter follow-up, which may in part be indicative of reverse causality.^11 220^ For some outcomes, stronger associations were also observed in studies that did not include adjustment for adiposity in their analytical models, which can be interpreted as either being prone to residual confounding or a more inclusive estimation of the total causal effect, if adiposity is considered a mediator between PA and the investigated health outcomes.

A strength of our study was the use of comprehensive exposure data harmonisation methods that enabled the inclusion of a larger evidence base. This also allowed us to study health associations over a wider range of the PA exposure and increased the density of data in the area of the dose–response curves with high non-linearity. Another strength is the use of more sophisticated meta-analytical methods that allow the shape of the dose–response association to vary across the exposure range, rather than linear models of transformed exposure or grouping doses within high versus low PA or multiple exposure intervals. We improved the placement of spline knots compared with our previous work^13^ by considering the distribution of person-years instead of non-weighted associations across the PA range, acknowledging the paucity of data at higher volumes of PA.

Our study also has limitations. There are levels of the PA exposure for which health associations are not reported in many of the original studies and hence there is still substantial uncertainty at those levels in the meta-analyses for some outcomes. Many of the included studies relied on self-reported questionnaires without validation or calibration data that could be used in the exposure harmonisation. In the exposure data harmonisation procedures, it was also sometimes necessary to make assumptions about parameters of PA, such as intensity and duration, where these were not explicitly reported. Nonetheless, our sensitivity analyses demonstrated the robustness of our results to the assumptions made. Measurement error is likely to lead to an underestimation of the true association between PA and the various outcomes, as demonstrated by the stronger associations observed in studies using device-based measures of PA.^221^ Regression dilution bias, particularly in studies with longer follow-up times, might also have affected the associations observed,^220^ which can be mitigated by repeated PA measures over time. Misclassification of some outcomes will have occurred in original studies as ascertainment of outcomes may not be similarly accurate across all outcomes and studies. Residual confounding and reverse causation could remain. To mitigate confounding, the most comprehensively adjusted risk estimates were used in this meta-analysis. However, the level of covariate adjustment and the degree to which these covariates effectively control for confounding were not consistent across all studies. We excluded studies with less than 3 years of follow-up to mitigate reverse causation.^220^ Although the bias from reverse causation diminishes with longer periods of follow-up, changes in PA level over time could be possible.^11 222^ Our estimates of population impact are based on the assumptions that the dose–response relationships are causal and that the prevalence of PA in our cohorts is representative of the wider population. The time lag between searches and publication of our results is longer than typical due to the scale of this review. We did not use validated tools to assess risk of bias or certainty of evidence. Nonetheless, we assessed six critical potential sources of bias for observational studies in PA and health outcomes and quantified their impacts on our results. Aspects included in other tools but not quantified by us, such as methods to measure exposure and outcome and attempt to control for established confounding factors (eg, sex and age), were not materially different across the included studies and hence it was not possible to assess their impact. Our analyses also cover aspects that are important for judging the confidence in the evidence base, such as the dose–response gradient, precision of the effect estimates, risk of bias assessment and subgroup analysis to investigate the effect of study-level confounders.

Our findings support the current PA recommendations of 150–300 min/week of moderate aerobic PA (or 75–150 min/week of vigorous aerobic PA, or an equivalent combination of moderate and vigorous activities),^21^ in that this exposure level generally seems to equate to maximum or near-to-maximal benefits. However, the dose–response associations also demonstrate that appreciable health benefits can be gained from 75 min/week or less of moderate activity (ie, half the recommended minimum level). Thus, our findings support the recent change in public health messaging to ‘doing some PA is better than doing none’, and suggest that the emphasis on threshold-based recommendations could be further reduced.

It should be noted that our exposure estimates are derived from a variety of self-reported questionnaires that capture mostly moderate and vigorous activities. These exposure estimates differ from those derived using device-based measures, which also record light-intensity and intermittent activities that are more difficult to recall.^221 223 224^ Self-report and device-based measures are therefore complementary but not interchangeable,^224^ an important consideration when formulating public health messages.

Future research should investigate the reasons for the apparent inconsistencies in dose–response associations between composite and individual disease endpoints. Although our risk of bias and sensitivity analyses showed robustness to the approaches we took during data handling and dose harmonisation, future studies could quantify methodological uncertainties (eg, inaccuracies in exposure assessment) and propagate them in the aetiological analyses to provide more realistic uncertainty ranges for the dose–response associations. The evidence base was weaker for very high volumes of activity and further research is required to ascertain the shape of associations more reliably at the higher end of the PA continuum.

## Conclusion

We found evidence of dose-dependent associations between increasing non-occupational PA and a wide range of mortality, CVD and cancer outcomes. The strongest associations were observed for all-cause and CVD mortality, with weaker associations for the incidence of cancer, including variation by site. Appreciable population health benefits might be gained from increasing PA levels of people who are inactive to just half the current health recommendations, with further benefits for all reaching at least the recommended level and smaller additional benefits beyond that.

**What is already known**

- Higher levels of physical activity are associated with lower rates of premature death and chronic disease outcomes.
- The shape of the dose–response association has been more difficult to determine and has not been established for a range of chronic diseases.
- Accurate estimation of the dose–response association between physical activity and disease outcomes is needed to assess the disease burden of physical inactivity and the potential impact of changes to population levels of physical activity.

**What are the findings?**

- Our findings suggest an appreciably lower risk of mortality, cardiovascular diseases and cancers from the equivalent of 75 min/week or less of moderate-intensity aerobic physical activity (ie, half the recommended minimum levels).
- Our results include the first dose–response meta-analysis of nine site-specific cancers: bladder, esophageal, gastric cardia, head and neck, kidney, liver, lung, myeloid leukaemia and myeloma.
- One in 10 premature deaths could have been prevented if everyone achieved even half the recommended level of physical activity.
