# Supplementary material for: Non-occupational physical activity and risk of cardiovascular disease, cancer and mortality outcomes: a dose–response meta-analysis of large prospective studies
Source: Br J Sports Med. 2023 Feb 28;57(15):979–89. doi: 10.1136/bjsports-2022-105669 (PMC10423495; doi:10.1136/bjsports-2022-105669)
Supplement: Supplementary data [file bjsports-2022-105669supp001.pdf]

Paper: Non-occupational physical activity and risk of cardiovascular disease, cancer, and mortality outcomes: a dose-response meta-analysis of large prospective studies

Authors: Leandro Garcia, Matthew Pearce, Ali Abbas, Alexander Mok, Tessa Strain, Sara Ali, Alessio Crippa, Paddy C. Dempsey, Rajna Golubic, Paul Kelly, Yvonne Laird, Eoin McNamara, Samuel Moore, Thiago Herick de Sa, Andrea D. Smith, Katrien Wijndaele, James Woodcock, Søren Brage

Table of Contents

eMethods 1: study inclusion and exclusion criteria ... 2

eMethods 2: search strings ... 3

eMethods 3: article selection criteria when multiple reports on the same cohort and outcome were available ... 5

eMethods 4: data imputation procedures ... 6

eMethods 5: estimating the resting component of energy expenditure to convert MET to mMET ... 9

eMethods 6: potential population impact fraction (PIF) formula ... 10

eFigure 1: study screening and selection flowchart ... 11

eResults 1: meta-analysis of the 11 studies that reported all-cause, cardiovascular and cancer mortality risk ... 12

eResults 2: sex-stratified results ... 13

eResults 3: risk of bias assessment ... 14

eResults 4: sensitivity analysis results ... 17

eReferences ... 20

eMethods 1: study inclusion and exclusion criteria

Include	Exclude
**Participants**	
Human adults (18+ years of age)	Cohorts of institutionalised adults, medical patients (i.e., in secondary or tertiary prevention) or high-risk populations (e.g., hypertension, diabetes - except conditions inevitable with ageing, as post-menopause)
**Exposure**	
Volume (marginal MET-h/week) of non-occupational physical activity – this includes leisure-time physical activity alone, combined with other non-occupational physical activity domains (e.g., transport and/or domestic activity), or combined with specific types of activity such as walking. Physical activity volume can be either self-reported or measured by devices and must be reported in at least three exposure levels	Measures of physical activity from which occupational activity cannot be factored out; individual domains of physical activity which occur outside of work but do not include leisure-time activity (e.g., travel physical activity only); other risk factors either alone or combined with physical activity
**Comparators**	
Lower volumes of physical activity (as defined in 'Interventions')	
**Outcomes**	
1) All-cause mortality<br>2) Cardiovascular diseases (fatal and non-fatal):<br>a. Total CVD (I00-I99)*<br>b. Stroke (I60-I66)<br>c. Heart failure (I50, I11.0, I13.0, I13.2)<br>d. Coronary heart disease (I20-I25)<br>3) Cancers** (fatal and non-fatal):<br>a. Total cancer (C00-D49)<br>b. Breast (C50)<br>c. Endometrial (C54)<br>d. Lung (C34)<br>e. Colon (C18)<br>f. Esophageal (C15)<br>g. Liver (C22-C24)<br>h. Kidney (C64-C65)<br>i. Gastric cardia (C16.0)<br>j. Myeloid leukemia (C92)<br>k. Myeloma (C90)<br>l. Head and neck (C76.0)<br>m. Rectum (C20)<br>n. Bladder (C67)<br>o. Prostate (C61)<br>p. Malignant melanoma (C43)<br>Cases ascertained via health, death, cancer, hospital or insurance registers; death certificates; participants and their next-of-kin	Less than three years of follow-up period
**Study design**	
General population prospective cohort studies (including case-cohort and nested case-control studies) with sample size larger than 10,000 participants; results must be reported either as hazard ratios, relative risks or odds ratios with confidence intervals	Other study designs (e.g., experimental or cross-sectional), even when nested in prospective cohort studies

* Diseases coded using the International statistical classification of diseases and related health problems, 10th revision.[1]
** Cancers associated with leisure-time physical activity in Moore et al.[2]

- Type of documents: peer-reviewed articles published in academic journals.
- No limits on language and date of publication.

eMethods 2: search strings

Pubmed

((physical activit*[Title/Abstract]) OR (physical inactivity[Title/Abstract]) OR (exercise[Title/Abstract]) OR (exercises[Title/Abstract]) OR (exercising[Title/Abstract]) OR (recreational activit*[Title/Abstract]) OR (sport[Title/Abstract]) OR (sports[Title/Abstract]) OR (active transport[Title/Abstract]) OR (active transportation[Title/Abstract]) OR (active commut*[Title/Abstract]) OR (active travel*[Title/Abstract]) OR (household activit*[Title/Abstract]) OR (housework[Title/Abstract]) OR (non-exercise activit*[Title/Abstract]) OR (nonexercise activit*[Title/Abstract]) OR (activities of daily living[Title/Abstract]) OR (bicycle[Title/Abstract]) OR (bicycling[Title/Abstract]) OR (bike[Title/Abstract]) OR (biking[Title/Abstract]) OR (walk[Title/Abstract]) OR (walking[Title/Abstract]) OR (run[Title/Abstract]) OR (running[Title/Abstract]) OR (jogging[Title/Abstract]) OR (swim[Title/Abstract]) OR (swimming[Title/Abstract]) OR (resistance training[Title/Abstract]) OR (weight lifting[Title/Abstract]) OR (physical fitness[Title/Abstract]) OR (physical endurance[Title/Abstract]) OR (activity energy expenditure[Title/Abstract]) OR (caloric expenditure[Title/Abstract]) OR (motor activity[Mesh:NoExp]) OR (exercise[Mesh:NoExp]) OR (human physical conditioning[Mesh]) OR (leisure activities[Mesh:NoExp]) OR (recreation[Mesh:NoExp]) OR (dancing[Mesh]) OR (gardening[Mesh]) OR (sports[Mesh]) OR (activities of daily living[Mesh:NoExp]))
AND
((mortality[Title/Abstract]) OR (cardiovascular disease*[Title/Abstract]) OR (acute coronary syndrome[Title/Abstract]) OR (myocardial infarction[Title/Abstract]) OR (myocardial ischemia[Title/Abstract]) OR (heart disease*[Title/Abstract]) OR (coronary disease*[Title/Abstract]) OR (coronary artery disease*[Title/Abstract]) OR (heart failure[Title/Abstract]) OR (cardiac failure[Title/Abstract]) OR (stroke[Title/Abstract]) OR (brain ischemia[Title/Abstract]) OR (cerebrovascular accident*[Title/Abstract]) OR (cerebrovascular disorder*[Title/Abstract]) OR (cerebrovascular disease*[Title/Abstract]) OR (intracranial arterial disease*[Title/Abstract]) OR (ganglia hemorrhage[Title/Abstract]) OR (ganglia haemorrhage[Title/Abstract]) OR (cerebral intraventricular hemorrhage[Title/Abstract]) OR (cerebral intraventricular haemorrhage[Title/Abstract]) OR (hypertensive intracranial hemorrhage[Title/Abstract]) OR (hypertensive intracranial haemorrhage[Title/Abstract]) OR (cerebral haemorrhage[Title/Abstract]) OR (cerebral hemorrhage[Title/Abstract]) OR (cancer[Title/Abstract]) OR (cancers[Title/Abstract]) OR (tumour[Title/Abstract]) OR (tumours[Title/Abstract]) OR (tumor[Title/Abstract]) OR (tumors[Title/Abstract]) OR (neoplasm[Title/Abstract]) OR (neoplasms[Title/Abstract]) OR (mortality[Mesh:NoExp]) OR (cause of death[Mesh]) OR (premature mortality[Mesh]) OR (cardiovascular diseases[Mesh:NoExp]) OR (heart diseases[Mesh:NoExp]) OR (myocardial ischemia[Mesh]) OR (heart failure[Mesh]) OR (cerebrovascular disorders[Mesh:NoExp]) OR (brain ischemia[Mesh]) OR (cerebral small vessel diseases[Mesh]) OR (intracranial arterial diseases[Mesh]) OR (basal ganglia hemorrhage [Mesh]) OR (cerebral intraventricular hemorrhage [Mesh]) OR (hypertensive intracranial hemorrhage [Mesh]) OR (stroke[Mesh]) OR (neoplasms[Mesh]))
AND
((cohort[Title/Abstract]) OR (cohorts[Title/Abstract]) OR (follow-up study[Title/Abstract]) OR (follow-up studies[Title/Abstract]) OR (prospective study[Title/Abstract]) OR (prospective studies[Title/Abstract]) OR (longitudinal study[Title/Abstract]) OR (longitudinal studies[Title/Abstract]) OR (cohort studies[Mesh:NoExp]) OR (follow-up studies[Mesh]) OR (longitudinal studies[Mesh:NoExp]) OR (prospective studies[Mesh]))

Garcia L, *et al. Br J Sports Med* 2023; 57:979–989. doi: 10.1136/bjsports-2022-105669

Scopus

(TITLE-ABS-KEY(“physical activit*”) OR TITLE-ABS-KEY(“physical inactivity”) OR TITLE-ABS-KEY(exercise) OR TITLE-ABS-KEY(exercises) OR TITLE-ABS-KEY(exercising) OR TITLE-ABS-KEY(“recreational activit*”) OR TITLE-ABS-KEY(sport) OR TITLE-ABS-KEY(sports) OR TITLE-ABS-KEY(“active transport”) OR TITLE-ABS-KEY(“active transportation”) OR TITLE-ABS-KEY(“active commut*”) OR TITLE-ABS-KEY(“active travel*”) OR TITLE-ABS-KEY(“household activit*”) OR TITLE-ABS-KEY(housework) OR TITLE-ABS-KEY(“non-exercise activit*”) OR TITLE-ABS-KEY(“nonexercise activit*”) OR TITLE-ABS-KEY(“activities of daily living”) OR TITLE-ABS-KEY(bicycle) OR TITLE-ABS-KEY(bicycling) OR TITLE-ABS-KEY(bike) OR TITLE-ABS-KEY(biking) OR TITLE-ABS-KEY(walk) OR TITLE-ABS-KEY(walking) OR TITLE-ABS-KEY(run) OR TITLE-ABS-KEY(running) OR TITLE-ABS-KEY(jogging) OR TITLE-ABS-KEY(swim) OR TITLE-ABS-KEY(swimming) OR TITLE-ABS-KEY(“resistance training”) OR TITLE-ABS-KEY(“weight lifting”) OR TITLE-ABS-KEY(“physical fitness”) OR TITLE-ABS-KEY(“physical endurance”) OR TITLE-ABS-KEY(“activity energy expenditure”) OR TITLE-ABS-KEY(“caloric expenditure”))

AND

(TITLE-ABS-KEY(mortality) OR TITLE-ABS-KEY(“cardiovascular disease*”) OR TITLE-ABS-KEY(“acute coronary syndrome”) OR TITLE-ABS-KEY(“myocardial infarction”) OR TITLE-ABS-KEY(“myocardial ischemia”) OR TITLE-ABS-KEY(“heart disease*”) OR TITLE-ABS-KEY(“coronary disease*”) OR TITLE-ABS-KEY(“coronary artery disease*”) OR TITLE-ABS-KEY(“heart failure”) OR TITLE-ABS-KEY(“cardiac failure”) OR TITLE-ABS-KEY(stroke) OR TITLE-ABS-KEY(“brain ischemia”) OR TITLE-ABS-KEY(“cerebrovascular accident*”) OR TITLE-ABS-KEY(“cerebrovascular disorder*”) OR TITLE-ABS-KEY(“cerebrovascular disease*”) OR TITLE-ABS-KEY(“intracranial arterial disease*”) OR TITLE-ABS-KEY(“ganglia hemorrhage") OR TITLE-ABS-KEY(“ganglia haemorrhage”) OR TITLE-ABS-KEY(“cerebral intraventricular hemorrhage”) OR TITLE-ABS-KEY(“cerebral intraventricular haemorrhage”) OR TITLE-ABS-KEY(“hypertensive intracranial hemorrhage") OR TITLE-ABS-KEY(“hypertensive intracranial haemorrhage") OR TITLE-ABS-KEY(“cerebral hemorrhage”) OR TITLE-ABS-KEY(“cerebral haemorrhage”) OR TITLE-ABS-KEY(cancer) OR TITLE-ABS-KEY(cancers) OR TITLE-ABS-KEY(tumour) OR TITLE-ABS-KEY(tumours) OR TITLE-ABS-KEY(tumor) OR TITLE-ABS-KEY(tumors) OR TITLE-ABS-KEY(neoplasm) OR TITLE-ABS-KEY(neoplasms))

AND

(TITLE-ABS-KEY(cohort) OR TITLE-ABS-KEY(cohorts) OR TITLE-ABS-KEY(“follow-up study”) OR TITLE-ABS-KEY(“follow-up studies”) OR TITLE-ABS-KEY(“prospective study”) OR TITLE-ABS-KEY(“prospective studies”) OR TITLE-ABS-KEY(“longitudinal study”) OR TITLE-ABS-KEY(“longitudinal studies”))

Web of Science

(TS=(“physical activit*”) OR TS=(“physical inactivity”) OR TS=(exercise) OR TS=(exercises) OR TS=(exercising) OR TS=(“recreational activit*”) OR TS=(sport) OR TS=(sports) OR TS=(“active transport”) OR TS=(“active transportation”) OR TS=(“active commut*”) OR TS=(“active travel*”) OR TS=(“household activit*”) OR TS=(housework) OR TS=(“non-exercise activit*”) OR TS=(“nonexercise activit*”) OR TS=(“activities of daily living”) OR TS=(bicycle) OR TS=(bicycling) OR TS=(bike) OR TS=(biking) OR TS=(walk) OR TS=(walking) OR TS=(run) OR TS=(running) OR TS=(jogging) OR TS=(swim) OR TS=(swimming) OR TS=(“resistance training”) OR TS=(“weight lifting”) OR TS=(“physical fitness”) OR TS=(“physical endurance”) OR TS=(“activity energy expenditure”) OR TS=(“caloric expenditure”))

AND

(TS=(mortality) OR TS=(“cardiovascular disease*”) OR TS=(“acute coronary syndrome”) OR TS=(“myocardial infarction”) OR TS=(“myocardial ischemia”) OR TS=(“heart disease*”) OR TS=(“coronary disease*”) OR TS=(“coronary artery disease*”) OR TS=(“heart failure”) OR TS=(“cardiac failure”) OR TS=(stroke) OR TS=(“brain ischemia”) OR TS=(“cerebrovascular accident*”) OR TS=(“cerebrovascular disorder*”) OR TS=(“cerebrovascular disease*”) OR TS=(“intracranial arterial disease*”) OR TS=(“ganglia hemorrhage") OR TS=(“ganglia haemorrhage”) OR TS=(“cerebral intraventricular hemorrhage”) OR TS=(“cerebral intraventricular haemorrhage”) OR TS=(“hypertensive intracranial hemorrhage") OR TS=(“hypertensive intracranial haemorrhage") OR TS=(“cerebral hemorrhage”) OR TS=(“cerebral haemorrhage”) OR TS=(cancer) OR TS=(cancers) OR TS=(tumour) OR TS=(tumours) OR TS=(tumor) OR TS=(tumors) OR TS=(neoplasm) OR TS=(neoplasms))

AND

(TS=(cohort) OR TS=(cohorts) OR TS=(“follow-up study”) OR TS=(“follow-up studies”) OR TS=(“prospective study”) OR TS=(“prospective studies”) OR TS=(“longitudinal study”) OR TS=(“longitudinal studies”))

Garcia L, *et al. Br J Sports Med* 2023; 57:979–989. doi: 10.1136/bjsports-2022-105669

eMethods 3: article selection criteria when multiple reports on the same cohort and outcome were available

The following criteria were used in the indicated hierarchy. That is, if two or more article were found and they balanced each other out in terms of the criteria (i.e., one was better for one criterion, but the other one for a second criterion), this hierarchy was then used to decide which article to select.

1) Lower risk of bias (exposure assessment, outcome ascertainment, exclusion of cases at baseline, exclusion of participants with relevant comorbidities at baseline or inclusion of baseline comorbidities as covariates in the regression model, and exclusion of new cases in the first years of follow-up).
2) Preferred measure of physical activity (e.g., MET-h/day rather than sessions).
3) More physical activity levels.
4) Separate results for men and women.
5) Longer follow-up period.

eMethods 4: data imputation procedures

CRITICAL INFORMATION (required to run the meta-analysis)

Number of individuals in each exposure category (required only when risk estimate presented as relative risk or odds ratio):

1) Estimated via proportional weighting using the following equation:

$$\frac{p_i}{P}S \quad (1)$$

Where p_i is person-years in the ith exposure category, P is total person-years, and S is total analytical sample size.

2) Or estimated via proportional weighting using the following steps (when person-years in each exposure category was not reported):

$$w_i = \frac{c_i}{(c_1 RR_i)} \quad (2)$$

$$s_1 = \frac{S}{\sum_{i=1}^{n} w_i} \quad (3)$$

$$s_i = w_i s_1 \quad (4)$$

Where w_i is the ratio between person-years in the ith exposure category and in the reference exposure category, c_i is cases in the ith exposure category (subscript 1 refers to the reference level), RR_i is minimally adjusted relative risk in the ith exposure category, s_i is number of individuals in the ith exposure category, and S is total analytical sample size.

Equations 3 and 5 derive from:

$$RR_i = \frac{\frac{c_i}{s_i}}{\frac{c_1}{s_1}} = \frac{c_i s_1}{c_1 s_i} \rightarrow s_i = \frac{c_i s_1}{c_1 RR_i} \rightarrow \frac{s_i}{s_1} = \frac{c_i}{c_1 RR_i} \quad (5)$$

Equation 4 derives from:

$$S = \sum_{i=1}^{n} s_i = \sum_{i=1}^{n} w_i s_1 \quad (6)$$

Once s_1 is found, it can be used in Equation 5 to estimate number of individuals in all other exposure categories.

3) Or authors contacted.

Person-years in each exposure category (required only when risk estimate presented as hazard ratio):

1) Estimated via proportional weighting using the following equation:

$$\frac{n_i}{S}P \tag{7}$$

Where n_i is number of individuals in the ith exposure category, S is total analytical sample size, and P is total person-years.

2) Or estimated via proportional weighting (when number of individuals in each exposure category was not reported) using Equations 3 to 5 substituting the number of individuals in the ith exposure category (s_i) and total analytical sample size (S) by person-years in the ith exposure category and total person-years, respectively.
3) Or authors contacted.

Cases in each exposure category:

1) Estimated via proportional weighting using the following steps:

$$c_{1_t} = \frac{RR_1}{\sum_{i=1}^{n} RR_i} C \tag{8}$$

$$r_1 = \frac{c_{1_t}}{n_1} \tag{9}$$

$$r_i = RR_i r_1 \tag{10}$$

$$c_i = n_i r_i \frac{C}{\sum_{i=1}^{n}(n_i r_i)} \tag{11}$$

Where c_i is cases in the ith exposure category (subscript 1 refers to the reference level; subscript t indicates temporary (non-corrected) number of cases), RR_i is risk estimate in the ith exposure category, C is total number of cases, r_i is risk in the ith exposure category, n_i is person-years (or number of individuals) in the ith exposure category. Equation 11 is also used to estimate the final number of cases in the reference level.

2) Or authors contacted.

BACKGROUND INFORMATION (obtained or estimated only when needed to impute at least one of the critical information)

Total analytical sample size:

1) Calculated via summation of the number of individuals in each exposure category.
2) Or reported in the article.
3) Or estimated dividing total person-years by mean follow-up.
4) Or authors contacted.

Mean follow-up:

1) Reported in the article (mean or median follow-up).
2) Or calculated by dividing total person-years by total analytical sample size.
3) Or reported study follow-up duration (e.g., 1990 to 2010 = 20 years), as an approximate surrogate.
4) Or authors contacted.

Total person-years:

1) Calculated via summation of person-years in each exposure category.
2) Or reported in the article.
3) Or estimated multiplying total analytical sample size by mean follow-up.
4) Or authors contacted.

Total number of cases:

1) Calculated via summation of cases in each exposure category.
2) Or reported in the article.
3) Or authors contacted.

Garcia L, *et al. Br J Sports Med* 2023; 57:979–989. doi: 10.1136/bjsports-2022-105669

eMethods 5: estimating the resting component of energy expenditure to convert MET to mMET

Total physical activity volume can be calculated by multiplying duration by the rate of energy expenditure (intensity). Rate of energy expenditure is often expressed in gross units of metabolic equivalent of task (MET), which includes both a resting (1 MET) and an activity component (the remainder, e.g., 3.5 METs for a 4.5 gross MET activity).

When publications report activity volume in total MET-hours (hours * MET) per week, it is necessary to obtain information on the duration of activities making up this total such that 1 MET can be subtracted for each hour to remove the resting component and give physical activity energy expenditure in marginalised MET (mMET).

In the example below, a study reports total physical activity volume as 9 MET-hours. Knowledge of the duration of activity of two hours is necessary to calculate and remove the resting component giving marginalised MET (mMET).

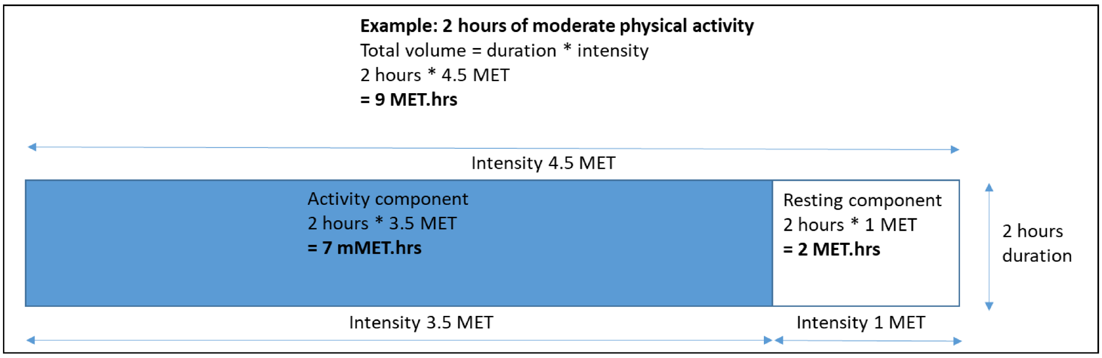

For studies that did not provide activity duration, a conversion equation from gross to marginal units is required. For the current analyses, we used a regression equation derived from the studies that provided both estimates, weighted for sample size per exposure category:

$$mMET\text{-}hours = 0.89 * MET\text{-}hours - 0.52 * MET\text{-}hours^{0.5} \quad (1)$$

The studies/cohorts contributing to this equation were:

Authors and year	Cohort	Participants
Leet et al.[3]	Aerobics Center Longitudinal Study	Men and women aged 20-82 years
Chomistek et al.[4]	Health Professionals Follow-up Study	Men aged 40-75 years
Chomistek et al.[5]*	Women's Genome Health Study	Women aged 45+ years
Wen et al.[6]	Taiwan Medical Screening Program	Men and women aged 20+ years
Mok et al.[7]	Severance Cohort Study	Men and women aged 30-93 years
Hu et al.[8]	Nurses' Health Study	Women aged 40-65 years
Besson et al.[9]*	EPIC-Norfolk	Men and women aged 40-79 years

* Identified by systematic literature review and used in equation derivation but not included in main analyses because full results were available in a parent study.

Garcia L, et al. Br J Sports Med 2023; 57:979–989. doi: 10.1136/bjsports-2022-105669

eMethods 6: potential population impact fraction (PIF) formula

The equation below has been adapted from Equation 7 in Zapata-Diomedi *et al*,[10] replacing prevalence for person-years.

$$PIF = \frac{\sum_{i=1}^{n} p_i RR_i - \sum_{i=1}^{n} p'_i RR_i}{\sum_{i=1}^{n} p_i RR_i}$$

Where p_i is the person-years in the ith exposure category, p'_i is the counterfactual, and RR_i is the relative risk of the exposed compared to the reference level of exposure.

Garcia L, *et al. Br J Sports Med* 2023; 57:979–989. doi: 10.1136/bjsports-2022-105669

eFigure 1: study screening and selection flowchart

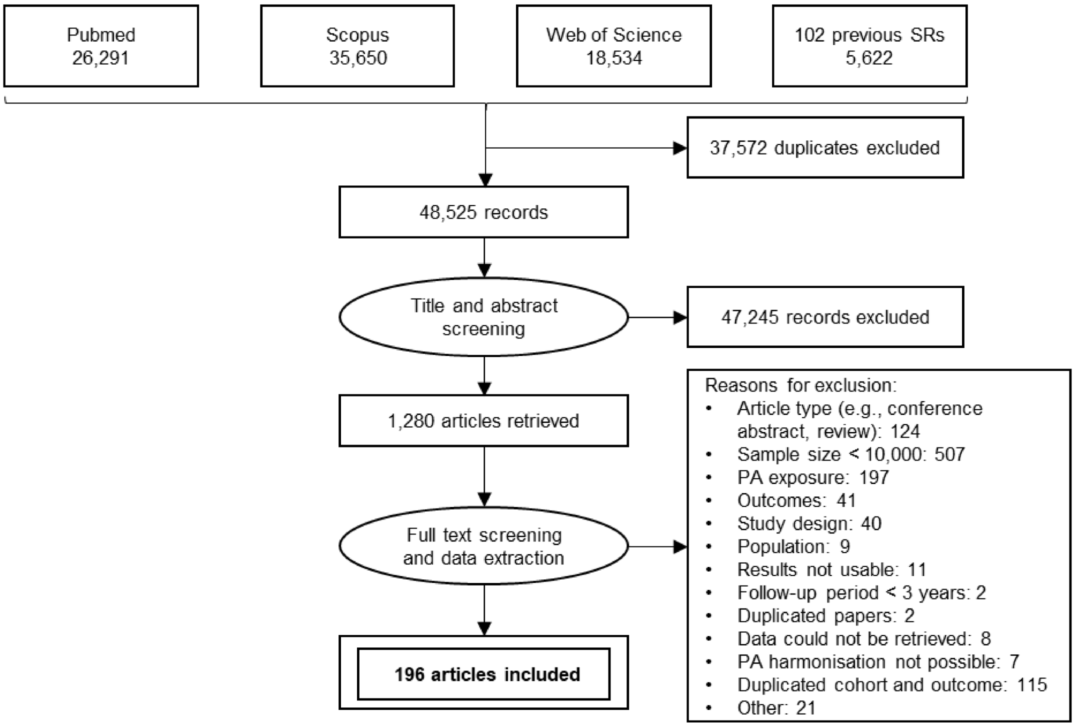

SR: systematic reviews. PA: physical activity.

Garcia L, *et al. Br J Sports Med* 2023; 57:979–989. doi: 10.1136/bjsports-2022-105669

eResults 1: meta-analysis of the 11 studies that reported all-cause, cardiovascular and cancer mortality risk

Outcomes	4.375 mMET-h/week RR (95% CI)	8.75 mMET-h/week RR (95% CI)	17.5 mMET-h/week RR (95% CI)
Subset of 11 studies[6, 11-20]			
All-cause mortality	0.81 (0.74 - 0.89)	0.67 (0.57 - 0.78)	0.61 (0.52 - 0.73)
Total CVD	0.77 (0.69 - 0.85)	0.67 (0.57 - 0.78)	0.61 (0.52 - 0.73)
Total cancer	0.89 (0.85 - 0.94)	0.84 (0.79 - 0.90)	0.81 (0.76 - 0.86)
All studies			
All-cause mortality	0.77 (0.73 - 0.80)	0.69 (0.65 - 0.73)	0.66 (0.62 - 0.70)
Total CVD	0.81 (0.77 - 0.85)	0.71 (0.66 - 0.77)	0.65 (0.60 - 0.71)
Total cancer	0.90 (0.88 - 0.93)	0.85 (0.81 - 0.89)	0.82 (0.78 - 0.86)

mMET = marginal metabolic equivalent of task; RR = relative risk; 95% CI = 95% confidence interval. CVD = cardiovascular disease. Reference group = 0 mMET-h/week.

Garcia L, *et al. Br J Sports Med* 2023; 57:979–989. doi: 10.1136/bjsports-2022-105669

eResults 2: sex-stratified results

Relative risk of mortality and incidence of cardiovascular diseases and cancers at three physical activity levels in relation to 0 mMET-h/week, by sex.

	4.375 mMET-h/week		8.75 mMET-h/week		17.5 mMET-h/week	
	Men	Women	Men	Women	Men	Women
**Outcomes**	**RR (95% CI)**	**RR (95% CI)**	**RR (95% CI)**	**RR (95% CI)**	**RR (95% CI)**	**RR (95% CI)**
Mortality						
All-cause mortality	0.88 (0.85 - 0.91)	0.84 (0.8 - 0.87)	0.80 (0.76 - 0.85)	0.71 (0.66 - 0.76)	0.75 (0.71 - 0.80)	0.71 (0.66 - 0.76)
Total CVD	0.87 (0.82 - 0.94)	0.81 (0.74 - 0.88)	0.80 (0.72 - 0.89)	0.67 (0.60 - 0.77)	0.75 (0.68 - 0.82)	0.67 (0.60 - 0.77)
Total cancer	0.90 (0.87 - 0.94)	0.95 (0.92 - 0.98)	0.86 (0.80 - 0.91)	0.88 (0.83 - 0.93)	0.84 (0.79 - 0.09)	0.88 (0.83 - 0.93)
CVD incidence (fatal and non-fatal events combined)						
Total CVD	0.89 (0.84 - 0.93)	0.84 (0.76 - 0.91)	0.81 (0.75 - 0.88)	0.70 (0.62 - 0.79)	0.76 (0.71 - 0.82)	0.70 (0.62 - 0.79)
Coronary heart disease	0.92 (0.85 - 0.99)	0.84 (0.75 - 0.93)	0.88 (0.79 - 0.98)	0.73 (0.60 - 0.87)	0.86 (0.76 - 0.97)	0.73 (0.60 - 0.87)
Stroke	0.90 (0.86 - 0.94)	0.88 (0.79 - 0.97)	0.85 (0.79 - 0.91)	0.76 (0.66 - 0.88)	0.82 (0.76 - 0.89)	0.76 (0.66 - 0.88)
Cancer incidence (fatal and non-fatal events combined)						
Total cancer	0.95 (0.92 - 0.98)	0.98 (0.96 - 0.99)	0.92 (0.86 - 0.97)	0.93 (0.89 - 0.97)	0.89 (0.82 - 0.96)	0.93 (0.89 - 0.97)
Colon	0.91 (0.86 - 0.96)	0.97 (0.92 - 1.02)	0.85 (0.78 - 0.94)	0.93 (0.84 - 1.03)	0.81 (0.72 - 0.91)	0.93 (0.84 - 1.03)
Rectum	0.92 (0.80 - 1.07)	1.09 (0.90 - 1.33)	0.90 (0.74 - 1.1)	1.13 (0.73 - 1.76)	0.92 (0.76 - 1.11)	1.13 (0.73 - 1.76)

mMET = marginal metabolic equivalent of task; RR = relative risk; 95% CI = 95% confidence interval. CVD = cardiovascular disease.

Potential impact fraction of physical activity on mortality and incidence of cardiovascular diseases and cancers at three physical activity levels, by sex.

	4.375 mMET-h/week		8.75 mMET-h/week		17.5 mMET-h/week	
	Men	Women	Men	Women	Men	Women
**Outcomes**	**PIF (95% CI)**	**PIF (95% CI)**	**PIF (95% CI)**	**PIF (95% CI)**	**PIF (95% CI)**	**PIF (95% CI)**
Mortality						
All-cause mortality	3.74 (2.69 - 4.83)	5.43 (4.00 - 6.94)	7.39 (5.52 - 9.31)	9.82 (7.42 - 12.29)	10.69 (8.37 - 13.03)	12.30 (9.30 - 15.35)
Total CVD	3.16 (1.51 - 4.86)	6.15 (3.54 - 8.85)	7.78 (4.48 - 11.02)	12.83 (8.06 - 17.52)	11.82 (9.62 - 14.04)	15.65 (11.65 - 19.64)
Total cancer	2.72 (1.57 - 3.90)	1.56 (0.67 - 2.48)	5.13 (3.11 - 7.18)	3.51 (1.68 - 5.36)	6.21 (4.20 - 8.25)	6.27 (3.94 - 8.59)
CVD incidence (fatal and non-fatal events combined)						
Total CVD	2.78 (1.54 - 4.05)	4.95 (2.45 - 7.54)	6.64 (4.17 - 9.10)	10.4 (5.90 - 14.84)	10.06 (8.23 - 11.91)	13.65 (10.48 - 16.86)
Coronary heart disease	2.99 (0.38 - 5.72)	7.10 (2.81 - 11.68)	5.07 (0.74 - 9.44)	12.17 (4.98 - 19.42)	6.15 (1.21 - 11.06)	14.60 (6.11 - 22.94)
Stroke	3.21 (1.72 - 4.80)	4.71 (1.02 - 8.60)	6.12 (3.47 - 8.89)	8.90 (2.92 - 14.91)	7.93 (4.87 - 11.07)	12.79 (7.22 - 18.38)
Cancer incidence (fatal and non-fatal events combined)						
Total cancer	1.38 (0.40 - 2.41)	0.73 (0.28 - 1.19)	3.09 (0.94 - 5.29)	1.77 (0.72 - 2.84)	4.94 (1.63 - 8.21)	3.92 (1.81 - 6.04)
Colon	2.07 (0.78 - 3.48)	0.67 (-0.44 - 1.87)	4.13 (1.68 - 6.71)	1.39 (-0.80 - 3.68)	6.42 (3.07 - 9.85)	2.25 (-0.55 - 5.11)
Rectum	1.90 (-1.52 - 5.71)	-2.64 (-7.02 - 3.40)	3.10 (-2.79 - 9.21)	-4.66 (-10.03 - 2.64)	1.43 (-3.83 - 6.99)	-4.57 (-31.13 - 20.26)

mMET = marginal metabolic equivalent of task; PIF = potential impact fraction; 95% CI = 95% confidence interval. CVD = cardiovascular disease.

Garcia L, *et al. Br J Sports Med* 2023; 57:979–989. doi: 10.1136/bjsports-2022-105669

eResults 3: risk of bias assessment

For the risk of bias assessment, we classified the harmonisation methods in two ways. Firstly, we classified studies depending on whether we made assumptions about the occupational component of an aggregated PA exposure (occupational PA not included in exposure; or assumptions about occupational PA made). Secondly, we described the overall method of converting exposure data to mMET-h/week (measurement unit conversion from MET, kcal, or kJ data; calibration using validation studies; using assumptions on ≤ 2 of intensity, duration, or frequency; or conversion of non-numeric data using assumptions on intensity, duration, and frequency). Note that for all-cause mortality and CVD outcomes one study is missing because 8.75mMET-h/week was not included in the exposure range reported in the original publication.

All-cause mortality

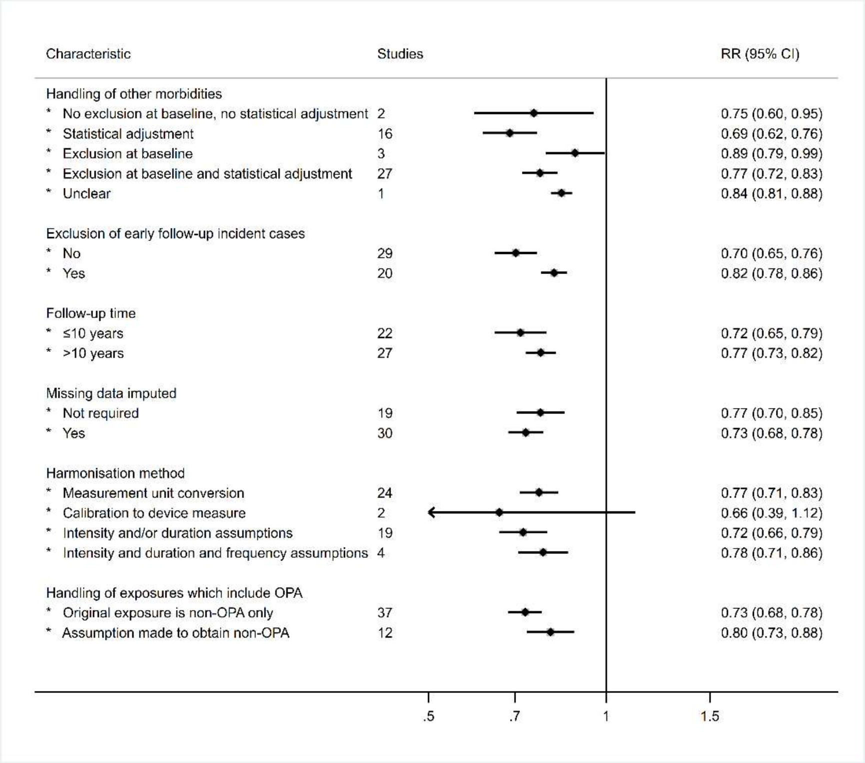

Garcia L, *et al. Br J Sports Med* 2023; 57:979–989. doi: 10.1136/bjsports-2022-105669

Total CVD mortality

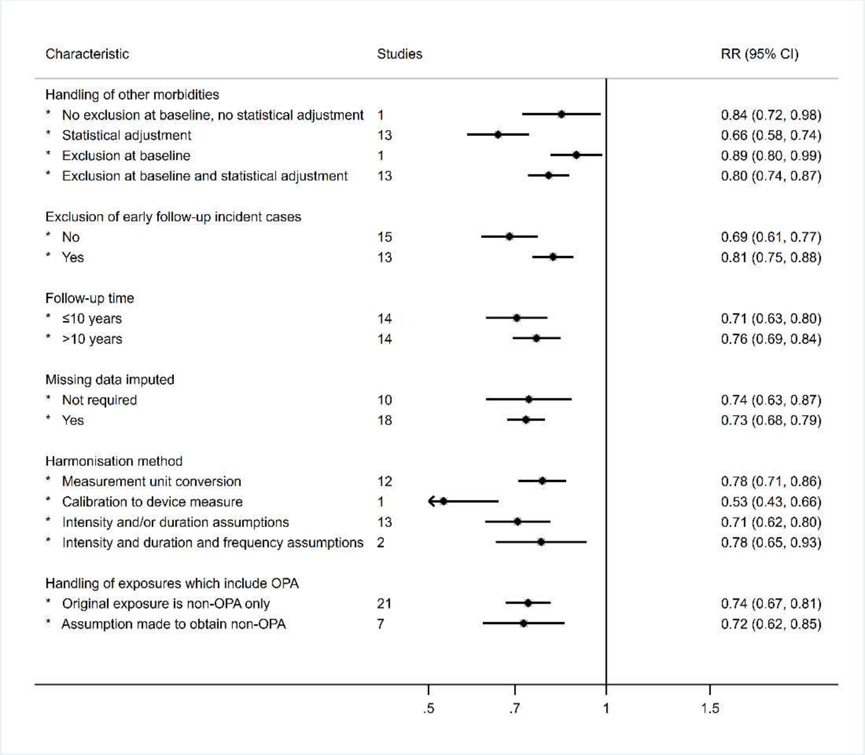

Total cancer mortality

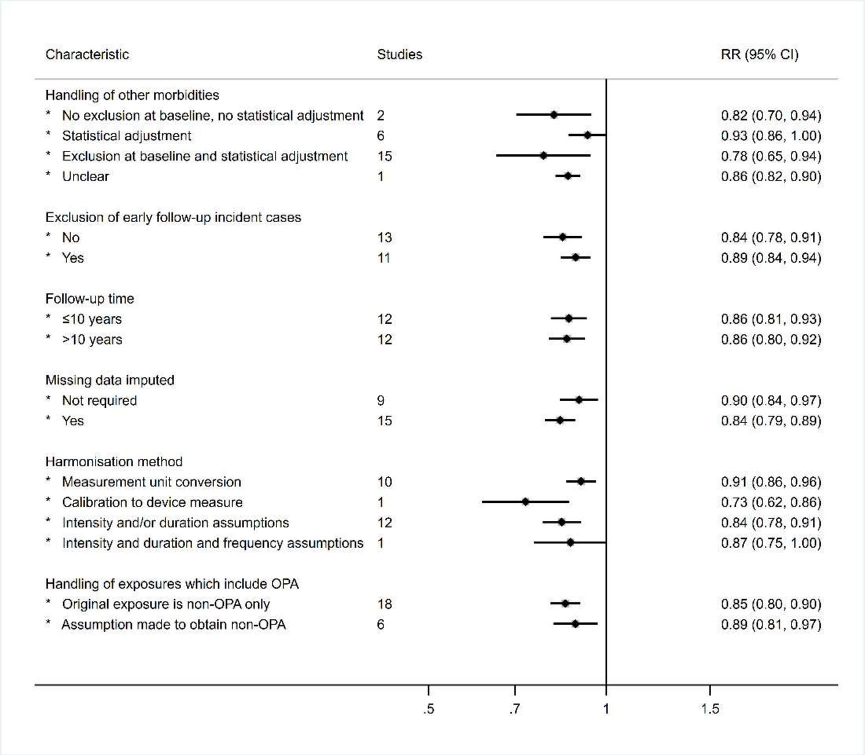

Total CVD incidence (fatal and non-fatal events combined)

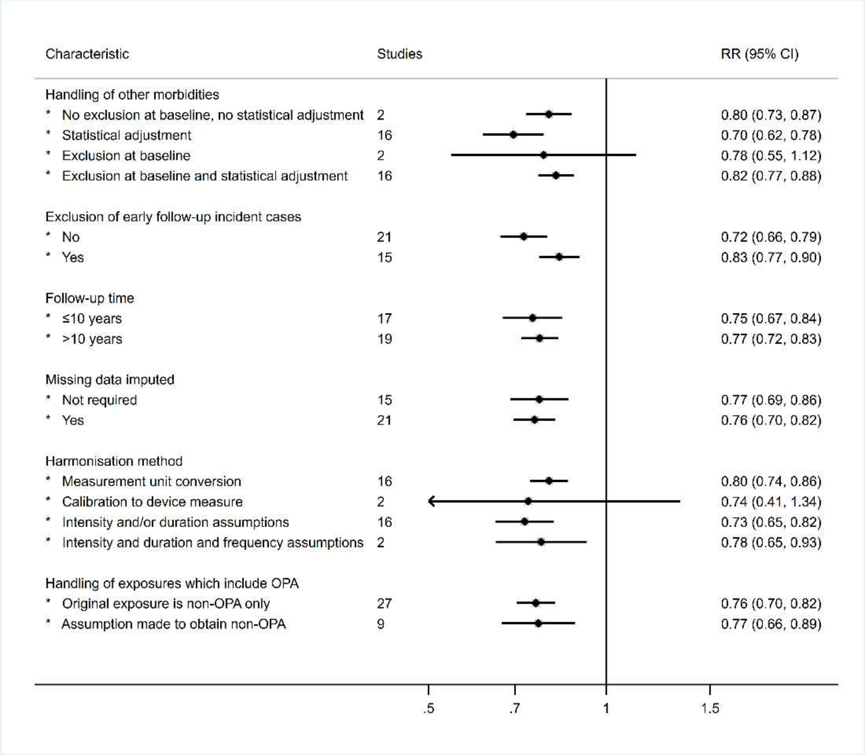

Total cancer incidence (fatal and non-fatal events combined)

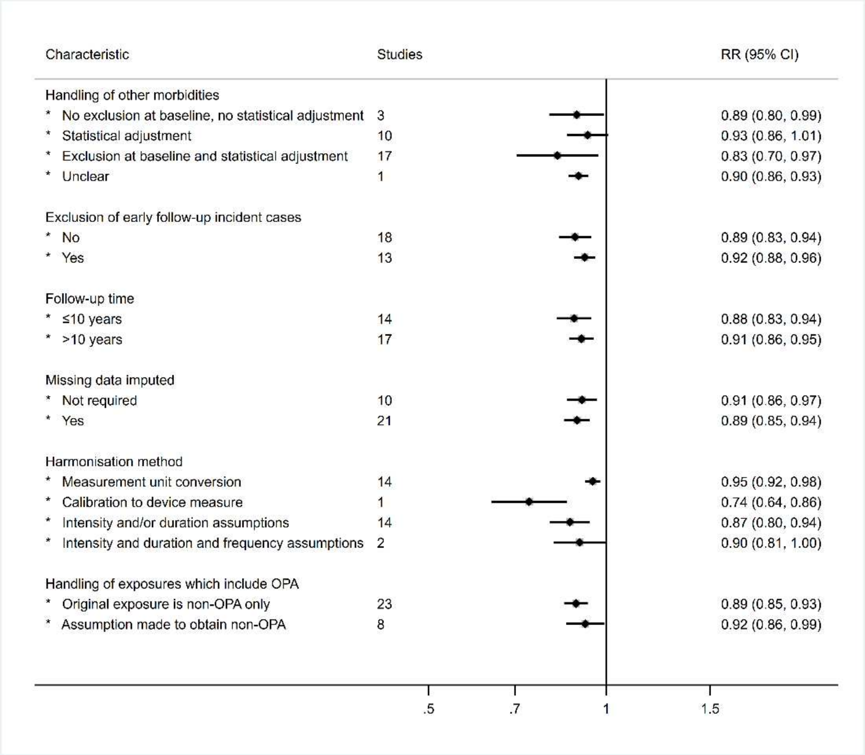

eResults 4: sensitivity analysis results

Relative risk of all-cause mortality and incidence of cardiovascular diseases and cancers at three physical activity levels in relation to 0 mMET-h/week, after alternative placement of knots (0th, 42.5th, and 85th percentile), alternative assumptions for physical activity intensity (1 mMET less to MPA and VPA) and session duration (0.5 hour rather than 0.75), and including only non-adiposity-adjusted models.

	Alternative knots position			Alternative PA intensity and duration assumptions			Non-adiposity-adjusted models only		
	**4.375**	**8.75**	**17.5**	**4.375**	**8.75**	**17.5**	**4.375**	**8.75**	**17.5**
**Outcomes**	**RR (95% CI)**	**RR (95% CI)**	**RR (95% CI)**	**RR (95% CI)**	**RR (95% CI)**	**RR (95% CI)**	**RR (95% CI)**	**RR (95% CI)**	**RR (95% CI)**
Mortality									
All-cause mortality	0.78 (0.75 - 0.81)	0.69 (0.65 - 0.74)	0.66 (0.62 - 0.71)	0.76 (0.72 - 0.79)	0.67 (0.63 - 0.72)	0.64 (0.60 - 0.69)	0.69 (0.63 - 0.76)	0.63 (0.57 - 0.71)	0.60 (0.54 - 0.68)
Total CVD	0.83 (0.79 - 0.87)	0.73 (0.67 - 0.79)	0.65 (0.60 - 0.71)	0.77 (0.73 - 0.82)	0.67 (0.61 - 0.74)	0.63 (0.57 - 0.69)	0.76 (0.68 - 0.84)	0.64 (0.54 - 0.75)	0.57 (0.47 - 0.69)
Total cancer	0.91 (0.89 - 0.94)	0.86 (0.82 - 0.89)	0.82 (0.78 - 0.86)	0.89 (0.86 - 0.92)	0.84 (0.80 - 0.88)	0.82 (0.79 - 0.86)	0.86 (0.81 - 0.92)	0.80 (0.74 - 0.87)	0.77 (0.71 - 0.84)
CVD incidence (fatal and non-fatal events combined)									
Total CVD	0.85 (0.81 - 0.88)	0.75 (0.70 - 0.80)	0.68 (0.63 - 0.73)	0.80 (0.76 - 0.84)	0.70 (0.65 - 0.76)	0.65 (0.60 - 0.71)	0.78 (0.72 - 0.84)	0.66 (0.59 - 0.75)	0.60 (0.51 - 0.69)
Coronary heart disease	0.88 (0.85 - 0.91)	0.80 (0.76 - 0.85)	0.74 (0.69 - 0.80)	0.82 (0.78 - 0.87)	0.77 (0.71 - 0.82)	0.74 (0.68 - 0.80)	0.84 (0.8 - 0.89)	0.76 (0.71 - 0.82)	0.71 (0.66 - 0.76)
Stroke	0.88 (0.85 - 0.91)	0.81 (0.76 - 0.85)	0.76 (0.71 - 0.81)	0.84 (0.81 - 0.88)	0.80 (0.76 - 0.84)	0.77 (0.72 - 0.84)	0.86 (0.82 - 0.91)	0.79 (0.74 - 0.85)	0.75 (0.70 - 0.81)
Heart failure	0.77 (0.63 - 0.94)	0.66 (0.48 - 0.91)	0.63 (0.42 - 0.94)	0.90 (0.86 - 0.95)	0.83 (0.76 - 0.91)	0.75 (0.66 - 0.86)	0.85 (0.79 - 0.93)	0.77 (0.68 - 0.86)	0.70 (0.63 - 0.78)
Cancer incidence (fatal and non-fatal events combined)									
Total cancer	0.94 (0.92 - 0.96)	0.90 (0.87 - 0.93)	0.85 (0.80 - 0.89)	0.91 (0.89 - 0.94)	0.87 (0.84 - 0.91)	0.85 (0.81 - 0.89)	0.89 (0.84 - 0.93)	0.83 (0.78 - 0.90)	0.81 (0.74 - 0.88)
Head and neck	0.77 (0.63 - 0.94)	0.66 (0.48 - 0.91)	0.63 (0.42 - 0.94)	0.72 (0.50 - 1.03)	0.67 (0.45 – 1.00)	0.63 (0.40 - 0.99)	0.72 (0.59 - 0.88)	0.63 (0.47 - 0.84)	0.61 (0.41 - 0.91)
Myeloid leukemia	0.85 (0.78 - 0.93)	0.77 (0.67 - 0.88)	0.74 (0.62 - 0.89)	0.77 (0.60 - 0.99)	0.75 (0.58 - 0.97)	0.73 (0.54 - 0.97)	-	-	-
Myeloma	0.84 (0.72 - 0.98)	0.77 (0.62 - 0.96)	0.76 (0.62 - 0.94)	0.83 (0.72 - 0.96)	0.75 (0.60 - 0.93)	0.76 (0.60 - 0.97)	-	-	-
Gastric cardia	0.89 (0.82 - 0.96)	0.80 (0.70 - 0.92)	0.73 (0.60 - 0.88)	0.86 (0.77 - 0.96)	0.78 (0.66 - 0.92)	0.73 (0.60 - 0.90)	0.90 (0.78 - 1.05)	0.84 (0.66 - 1.08)	0.79 (0.57 - 1.10)
Lung	0.90 (0.88 - 0.93)	0.85 (0.80 - 0.89)	0.82 (0.74 - 0.91)	0.87 (0.85 - 0.90)	0.83 (0.79 - 0.87)	0.84 (0.74 - 0.95)	0.83 (0.78 - 0.88)	0.76 (0.69 - 0.84)	0.78 (0.66 - 0.91)
Liver	0.90 (0.83 - 0.97)	0.82 (0.72 - 0.94)	0.75 (0.63 - 0.89)	0.88 (0.79 - 0.97)	0.82 (0.70 - 0.95)	0.78 (0.65 - 0.93)	0.89 (0.80 - 0.99)	0.82 (0.68 - 0.98)	0.76 (0.60 - 0.96)
Endometrial	0.95 (0.92 - 0.99)	0.91 (0.86 - 0.97)	0.87 (0.79 - 0.95)	0.93 (0.89 - 0.98)	0.89 (0.82 - 0.97)	0.86 (0.77 - 0.95)	0.89 (0.84 - 0.95)	0.81 (0.73 - 0.91)	0.73 (0.62 - 0.86)
Colon	0.97 (0.94 - 0.99)	0.94 (0.89 - 0.99)	0.91 (0.84 - 0.98)	0.95 (0.91 - 0.99)	0.92 (0.86 - 0.98)	0.90 (0.83 - 0.97)	0.97 (0.93 - 1.01)	0.94 (0.87 - 1.02)	0.92 (0.84 - 1.01)
Breast	0.97 (0.96 - 0.99)	0.95 (0.93 - 0.97)	0.92 (0.89 - 0.95)	0.96 (0.94 - 0.98)	0.93 (0.90 - 0.97)	0.91 (0.87 - 0.95)	0.97 (0.94 – 1.00)	0.95 (0.90 – 1.00)	0.92 (0.86 - 0.98)
Bladder	0.94 (0.86 - 1.02)	0.91 (0.81 - 1.02)	0.91 (0.81 - 1.02)	0.92 (0.82 - 1.02)	0.90 (0.81 - 1.01)	0.89 (0.76 - 1.06)	-	-	-

(continues)

Relative risk of all-cause mortality and incidence of cardiovascular diseases and cancers at three physical activity levels in relation to 0 mMET-h/week, after assigning alternative knots position (0th, 42.5th, and 85th percentile), using alternative assumptions for physical activity intensity (1 mMET less to MPA and VPA) and session duration (0.5 hour rather than 0.75), and including only non-adiposity-adjusted models (continuation).

	Alternative knots position			Alternative PA intensity and duration assumptions			Non-adiposity-adjusted models only		
	4.375	8.75	17.5	4.375	8.75	17.5	4.375	8.75	17.5
**Outcomes**	**RR (95% CI)**	**RR (95% CI)**	**RR (95% CI)**	**RR (95% CI)**	**RR (95% CI)**	**RR (95% CI)**	**RR (95% CI)**	**RR (95% CI)**	**RR (95% CI)**
Rectum	0.97 (0.93 - 1.01)	0.95 (0.89 - 1.03)	0.96 (0.87 - 1.05)	0.95 (0.9 – 1.0)	0.94 (0.87 - 1.01)	0.96 (0.89 - 1.04)	0.90 (0.84 - 0.97)	0.87 (0.78 - 0.96)	0.88 (0.79 - 0.98)
Esophageal	0.98 (0.91 - 1.05)	0.96 (0.85 - 1.08)	0.95 (0.80 - 1.12)	0.95 (0.85 - 1.07)	0.93 (0.78 - 1.11)	0.94 (0.77 - 1.13)	0.91 (0.78 - 1.06)	0.86 (0.67 - 1.11)	0.85 (0.63 - 1.16)
Prostate	1.00 (0.99 - 1.02)	1.01 (0.98 - 1.03)	1.01 (0.97 - 1.05)	1.01 (0.99 - 1.03)	1.01 (0.98 - 1.05)	1.01 (0.96 - 1.05)	1.01 (0.98 - 1.04)	1.02 (0.97 - 1.07)	1.02 (0.95 - 1.10)
Kidney	1.02 (0.94 - 1.11)	1.04 (0.91 - 1.19)	1.05 (0.88 - 1.26)	1.03 (0.90 - 1.16)	1.04 (0.88 - 1.22)	1.06 (0.84 - 1.33)	0.88 (0.79 - 0.98)	0.82 (0.71 - 0.96)	0.79 (0.68 - 0.93)

mMET = marginal metabolic equivalent of task; RR = relative risk; 95% CI = 95% confidence interval. CVD = cardiovascular disease.

Potential impact fraction of physical activity on all-cause mortality and incidence of cardiovascular diseases and cancers at three physical activity levels (in mMET-h/wk) after alternative placement of knots (0th, 42.5th, and 85th percentile), alternative assumptions for physical activity intensity (1 mMET less to MPA and VPA) and session duration (0.5 hour rather than 0.75), and including only non-adiposity-adjusted models.

	Alternative knots position			Alternative PA intensity and duration assumptions			Non-adiposity-adjusted models only		
	4.375	8.75	17.5	4.375	8.75	17.5	4.375	8.75	17.5
**Outcomes**	**PIF (95% CI)**	**PIF (95% CI)**	**PIF (95% CI)**	**PIF (95% CI)**	**PIF (95% CI)**	**PIF (95% CI)**	**PIF (95% CI)**	**PIF (95% CI)**	**PIF (95% CI)**
Mortality									
All-cause mortality	10.11 (8.32 - 11.91)	15.66 (13.16 - 18.15)	18.75 (16.09 - 21.39)	10.73 (8.88 - 12.62)	17.08 (14.36 - 19.77)	20.05 (17.01 - 23.04)	13.98 (10.35 - 17.65)	18.49 (14.15 - 22.8)	21.99 (17.4 - 26.5)
Total CVD	6.02 (4.42 - 7.69)	12.25 (9.37 - 15.17)	17.05 (13.92 - 20.19)	8.00 (5.89 - 10.20)	15.08 (11.52 - 18.68)	18.66 (14.79 - 22.53)	7.39 (4.41 - 10.67)	13.83 (8.65 - 19.19)	18.33 (12.26 - 24.43)
Total cancer	3.68 (2.54 - 4.86)	7.05 (5.10 – 9.00)	9.29 (7.36 - 11.22)	4.52 (3.13 - 5.94)	7.71 (5.68 - 9.75)	9.00 (7.20 - 10.80)	7.22 (4.07 - 10.46)	11.37 (7.09 - 15.68)	13.73 (9.38 - 18.06)
CVD incidence (fatal and non-fatal events combined)									
Total CVD	5.29 (3.93 - 6.70)	10.86 (8.43 - 13.31)	15.42 (13.01 - 17.85)	6.84 (5.10 - 8.63)	13.16 (10.24 - 16.1)	16.94 (14.07 - 19.82)	6.43 (4.19 - 8.88)	12.4 (8.41 - 16.54)	17.18 (12.36 - 22.06)
Coronary heart disease	5.51 (3.95 - 7.11)	10.28 (7.61 - 12.96)	13.58 (10.51 - 16.64)	7.93 (5.58 - 10.32)	12.13 (8.93 - 15.33)	14.31 (10.43 - 18.14)	6.26 (4.30 - 8.27)	11.28 (8.23 - 14.33)	14.97 (12.06 - 17.87)
Stroke	5.53 (3.96 - 7.15)	9.77 (7.31 - 12.24)	12.37 (9.63 - 15.1)	7.20 (5.33 - 9.10)	10.40 (8.12 - 12.69)	12.66 (8.46 - 16.77)	5.13 (3.29 - 7.03)	9.31 (6.51 - 12.14)	12.25 (9.45 - 15.08)
Heart failure	2.23 (0.80 - 3.89)	4.63 (1.90 - 7.66)	7.86 (4.31 - 11.68)	2.36 (1.15 - 3.74)	5.00 (2.49 - 7.75)	9.43 (4.94 - 14.10)	3.34 (1.34 - 5.78)	6.25 (3.09 - 9.90)	9.21 (5.86 - 12.84)
Cancer incidence (fatal and non-fatal events combined)									
Total cancer	2.53 (1.66 - 3.42)	5.22 (3.55 - 6.90)	7.80 (5.65 - 9.93)	3.48 (2.33 - 4.66)	6.56 (4.57 - 8.55)	8.57 (6.31 - 10.81)	5.67 (3.24 - 8.18)	9.12 (5.51 - 12.76)	11.12 (6.91 - 15.33)
Head and neck	10.59 (2.10 - 19.96)	17.46 (3.68 - 30.98)	19.83 (1.23 - 36.55)	13.82 (-1.07 - 28.5)	18.21 (1.05 - 34.26)	23.04 (2.16 - 41.14)	11.66 (4.19 - 19.84)	18.82 (6.67 - 30.81)	20.07 (0.44 - 37.22)
Myeloid leukemia	4.93 (0.68 - 9.70)	7.08 (1.65 - 12.94)	7.57 (2.23 - 13.33)	6.67 (0.40 - 13.47)	8.33 (1.91 - 15.25)	10.22 (1.38 - 19.18)	-	-	-
Myeloma	3.34 (0.90 - 6.26)	6.76 (1.90 - 12.09)	8.30 (1.91 - 14.99)	3.71 (0.79 - 7.23)	7.79 (1.75 - 14.37)	6.57 (-0.43 - 14.07)	-	-	-

(continues)

BMJ Publishing Group Limited (BMJ) disclaims all liability and responsibility arising from any reliance placed on this supplemental material which has been supplied by the author(s)

Potential impact fraction of physical activity on all-cause mortality and incidence of cardiovascular diseases and cancers at three physical activity levels (in mMET-h/wk) after assigning alternative knots position (0th, 42.5th, and 85th percentile), using alternative assumptions for physical activity intensity (1 mMET less to MPA and VPA) and session duration (0.5 hour rather than 0.75), and including only non-adiposity-adjusted models (continuation).

	Alternative knots position			Alternative PA intensity and duration assumptions			Non-adiposity-adjusted models only		
	**4.375**	**8.75**	**17.5**	**4.375**	**8.75**	**17.5**	**4.375**	**8.75**	**17.5**
**Outcomes**	**PIF (95% CI)**	**PIF (95% CI)**	**PIF (95% CI)**	**PIF (95% CI)**	**PIF (95% CI)**	**PIF (95% CI)**	**PIF (95% CI)**	**PIF (95% CI)**	**PIF (95% CI)**
Gastric cardia	1.95 (0.58 - 3.68)	4.77 (1.58 - 8.57)	6.77 (2.55 - 11.63)	2.45 (0.64 - 4.78)	5.40 (1.56 – 10.00)	7.29 (2.34 - 13.03)	1.13 (-0.42 - 3.29)	3.17 (-1.19 - 8.63)	5.12 (-1.81 - 13.09)
Lung	4.39 (3.16 - 5.63)	7.26 (5.10 - 9.4)	8.54 (3.04 - 13.98)	5.88 (4.05 - 7.37)	9.56 (6.13 - 12.49)	8.44 (-0.66 - 17.5)	8.01 (4.69 - 11.23)	12.27 (6.66 - 17.64)	10.52 (0.77 - 20.21)
Liver	3.49 (0.58 - 6.71)	6.75 (1.44 - 12.31)	10.27 (3.26 - 17.31)	4.59 (0.90 - 8.59)	8.37 (2.11 - 14.76)	11.07 (3.45 - 18.63)	4.62 (0.24 - 9.54)	8.72 (0.79 - 16.97)	12.86 (2.32 - 23.21)
Endometrial	1.10 (0.26 - 2.02)	2.62 (0.76 - 4.60)	4.54 (1.88 - 7.28)	1.50 (0.37 - 2.73)	3.66 (1.07 - 6.38)	5.91 (2.17 - 9.72)	2.23 (0.89 - 3.77)	5.66 (2.38 - 9.23)	10.82 (4.99 - 16.76)
Colon	0.70 (0.11 - 1.33)	1.69 (0.33 - 3.12)	2.96 (0.92 - 5.05)	0.94 (0.19 - 1.74)	2.12 (0.49 - 3.82)	3.73 (1.24 - 6.25)	0.45 (-0.15 - 1.11)	1.19 (-0.32 - 2.79)	2.29 (-0.06 - 4.70)
Breast	0.69 (0.32 - 1.07)	1.62 (0.79 - 2.46)	3.23 (1.85 - 4.63)	1.13 (0.53 - 1.75)	2.44 (1.21 - 3.69)	4.09 (2.35 - 5.83)	0.79 (-0.05 - 1.67)	1.78 (0.10 - 3.50)	3.68 (1.45 - 5.94)
Bladder	1.91 (-0.54 - 4.52)	2.98 (-0.24 - 6.33)	3.11 (0.02 - 6.35)	2.72 (-0.64 - 6.23)	3.70 (0.16 - 7.39)	4.59 (-3.62 - 12.48)	-	-	-
Rectum	0.77 (-0.15 - 1.75)	1.43 (-0.45 - 3.36)	0.52 (-1.74 - 2.82)	1.30 (-0.09 - 2.74)	2.07 (-0.49 - 4.65)	0.02 (-2.60 - 2.67)	2.07 (0.53 - 3.73)	3.74 (0.90 - 6.66)	2.69 (-0.19 - 5.66)
Esophageal	0.68 (-0.95 - 2.68)	1.38 (-2.06 - 5.32)	1.57 (-3.05 - 6.67)	1.02 (-1.34 - 3.88)	1.87 (-2.65 - 6.95)	1.58 (-3.44 - 7.13)	1.86 (-1.08 - 5.71)	3.7 (-2.48 - 10.93)	3.96 (-4.07 - 12.86)
Prostate	-0.10 (-0.48 - 0.29)	-0.21 (-1.10 - 0.71)	-0.10 (-1.61 - 1.42)	-0.16 (-0.64 - 0.35)	-0.29 (-1.36 - 0.81)	-0.11 (-1.75 - 1.54)	-0.23 (-0.90 - 0.48)	-0.5 (-2.02 - 1.08)	-0.65 (-3.10 - 1.85)
Kidney	-0.65 (-3.69 - 2.81)	-1.23 (-6.39 - 4.34)	-1.91 (-9.20 - 5.63)	-0.95 (-5.25 - 3.85)	-1.78 (-8.56 - 5.40)	-3.06 (-15.89 - 9.34)	5.61 (0.92 - 10.50)	9.24 (2.27 - 16.16)	11.73 (4.40 - 18.93)

mMET = marginal metabolic equivalent of task; PIF = potential impact fraction; 95% CI = 95% confidence interval. CVD = cardiovascular disease.

eReferences

1. World Health Organization. *International statistical classification of diseases and related health problems, 10th revision - 2018 edition*. World Health Organization; 2018.
2. Moore SC, Lee IM, Weiderpass E, et al. Association of leisure-time physical activity with risk of 26 types of cancer in 1.44 million adults. *JAMA Intern Med*. 2016;176(6):816-825.
3. Lee DC, Sui X, Ortega FB, et al. Comparisons of leisure-time physical activity and cardiorespiratory fitness as predictors of all-cause mortality in men and women. *Br J Sports Med*. 2011;45(6):504-510.
4. Chomistek AK, Cook NR, Flint AJ, Rimm EB. Vigorous-intensity leisure-time physical activity and risk of major chronic disease in men. *Med Sci Sports Exerc*. 2012;44(10):1898-1905.
5. Chomistek AK, Chasman DI, Cook NR, Rimm EB, Lee IM. Physical activity, genes for physical fitness, and risk of coronary heart disease. *Med Sci Sports Exerc*. 2013;45(4):691-697.
6. Wen CP, Wai JP, Tsai MK, et al. Minimum amount of physical activity for reduced mortality and extended life expectancy: a prospective cohort study. *Lancet*. 2011;378(9798):1244-53.
7. Mok Y, Won S, Kimm H, Nam C, Ohrr H, Jee SH. Physical activity level and risk of death: the Severance cohort study. *J Epidemiol*. 2012;22(6):494-500.
8. Hu FB, Stampfer MJ, Colditz GA, et al. Physical activity and risk of stroke in women. *JAMA*. 2000;283(22):2961-2967.
9. Besson H, Ekelund U, Brage S, et al. Relationship between subdomains of total physical activity and mortality. *Med Sci Sports Exerc*. 2008;40(11):1909-1915.
10. Zapata-Diomedi B, Barendregt JJ, Veerman JL. Population attributable fraction: names, types and issues with incorrect interpretation of relative risks. *Br J Sports Med*. 2018;52(4):212-213.
11. Zhou Y, Zhang R, Liu Y, et al. Association of regular physical activity with total and cause-specific mortality among middle-aged and older Chinese: a prospective cohort study. *Sci Rep*. 2017;7:39939.
12. Lee JY, Ryu S, Cheong E, Sung KC. Association of physical activity and inflammation with all-cause, cardiovascular-related, and cancer-related mortality. *Mayo Clin Proc*. 2016;91(12):1706-1716.
13. Liu Y, Wen W, Gao YT, et al. Level of moderate-intensity leisure-time physical activity and reduced mortality in middle-aged and elderly Chinese. *J Epidemiol Community Health*. 2018;72(1):13-20.
14. Matthews CE, Cohen SS, Fowke JH, et al. Physical activity, sedentary behavior, and cause-specific mortality in black and white adults in the Southern Community Cohort Study. *Am J Epidemiol*. 2014;180(4):394-405.
15. Hu G, Tuomilehto J, Silventoinen K, Barengo NC, Peltonen M, Jousilahti P. The effects of physical activity and body mass index on cardiovascular, cancer and all-cause mortality among 47 212 middle-aged Finnish men and women. *Int J Obes*. 2005;29(8):894-902.
16. Patel AV, Bernstein L, Deka A, et al. Leisure time spent sitting in relation to total mortality in a prospective cohort of US adults. *Am J Epidemiol*. 2010;172(4):419-429.
17. Schooling CM, Lam TH, Li ZB, et al. Obesity, physical activity, and mortality in a prospective chinese elderly cohort. *Arch Intern Med*. 2006;166(14):1498-504.
18. Wanner M, Tarnutzer S, Martin BW, et al. Impact of different domains of physical activity on cause-specific mortality: a longitudinal study. *Prev Med*. 2014;62:89-95.
19. Hamer M, De Oliveira C, Demakakos P. Non-exercise physical activity and survival: English Longitudinal Study of Ageing. *Am J Prev Med*. 2014;47(4):452-460.
20. Ueshima K, Ishikawa-Takata K, Yorifuji T, et al. Physical activity and mortality risk in the Japanese elderly. A cohort study. *Am J Prev Med*. 2010;38(4):410-418.
